# The Emerging World of Membrane Vesicles: Functional Relevance, Theranostic Avenues and Tools for Investigating Membrane Function

**DOI:** 10.3389/fmolb.2021.640355

**Published:** 2021-04-22

**Authors:** Aswin T. Srivatsav, Shobhna Kapoor

**Affiliations:** ^1^ Department of Chemistry, Indian Institute of Technology Bombay, Mumbai, India; ^2^ Wadhwani Research Center of Bioengineering, Indian Institute of Technology Bombay, Mumbai, India

**Keywords:** lipids, membrane vesicles, exosomes, membrane organization, host-pathogen interactions, lipid biomarkers, diagnosis, drug delivery

## Abstract

Lipids are essential components of cell membranes and govern various membrane functions. Lipid organization within membrane plane dictates recruitment of specific proteins and lipids into distinct nanoclusters that initiate cellular signaling while modulating protein and lipid functions. In addition, one of the most versatile function of lipids is the formation of diverse lipid membrane vesicles for regulating various cellular processes including intracellular trafficking of molecular cargo. In this review, we focus on the various kinds of membrane vesicles in eukaryotes and bacteria, their biogenesis, and their multifaceted functional roles in cellular communication, host-pathogen interactions and biotechnological applications. We elaborate on how their distinct lipid composition of membrane vesicles compared to parent cells enables early and non-invasive diagnosis of cancer and *tuberculosis*, while inspiring vaccine development and drug delivery platforms. Finally, we discuss the use of membrane vesicles as excellent tools for investigating membrane lateral organization and protein sorting, which is otherwise challenging but extremely crucial for normal cellular functioning. We present current limitations in this field and how the same could be addressed to propel a fundamental and technology-oriented future for extracellular membrane vesicles.

## Introduction

Lipids are fundamental components of the plasma membrane across various living forms and other cellular compartments such as nuclear membranes, Golgi, and endoplasmic reticulum. They are also the essential building blocks of cell-derived membrane vesicles. As part of these, lipids conform to diverse biological functions ranging from structural and signaling roles as well as by fine-tuning protein activity. Historically lipids have held a secondary place-next to proteins-in both basic and applied research attributed to the lack of techniques for their isolation, visualization, manipulation and quantitation. However, due to their undisputable involvement in a spectrum of diseases, there has been a surge in development of effective tools and methods for investigating lipids with unprecedented details.

Much we know about lipids and some of their presumed biological functions have come from the study of synthetic membranes with specific lipid composition due to the inability to study lipids in native environment. This is now fast changing with advent of super resolution fluorescence techniques, label-free mass imaging and lipidomics. However, the most impactful has been the discovery of membrane vesicles released from various kinds of cells (both eukaryotic, prokaryotic and archaea). These membrane vesicles are known by various names such as exosomes, apoptotic bodies, oncosomes derived from eukaryotes and outer membrane vesicles from bacterial species. These rather universal components have contributed significantly in enhancing our knowledge on the multi-level role of lipids in physiology, disease, and interestingly as theranostic modules, which were mostly only affiliated with proteins. The most commendable use of cell-derived vesicles in the form of giant plasma membrane vesicles (GPMVs), bacterial outer membrane vesicles (OMVs)-and lately giant endoplasmic reticulum vesicles (GERs)—has been to query the functional relevance for lipid phase separation or nanoclustering to form distinct membrane domains with selective recruitment of specific lipids and proteins that orchestrate various cellular processes, i.e., signaling (Baumgart et al., 2007; Grimmer and Bacia, 2020; Podkalicka and Blouin, 2020; Skinkle et al., 2020). Moreover, the lipid repertoire exclusive to these membrane vesicles is serving as attractive platform for developing selective and specific diagnosis markers against various diseases such as cancer, neurodegeneration, and infectious diseases.

Aligning with the varied roles of lipids within membrane vesicles, here, we review the latest development in this field. We elaborate on the various kinds of membrane vesicles and their biogenesis, comment on their composition and subsequently associated physical properties and how that is being exploited for early diagnosis in various diseases. The recent therapeutic prospects of membrane vesicles as delivery vehicles are discussed. We provide the breath of successful vaccine campaigns that have emerged from bacterial membrane vesicles and their constituent lipids components. Finally, we detail how the structural and compositional heterogeneity of various kinds of membrane vesicles are enabling exquisite insights into the functional roles of lipids in dictating membrane and protein function in eukaryotic and bacterial cells.

## Membrane Vesicles–Lipid Vesicular Assemblies Spanning the Tree of Life

Membrane vesicles are nano- or micrometer-sized membrane-bound lipid vesicles released from cells for systemic delivery of various kinds of molecular cargoes such as nucleic acids, lipids sugars and proteins to recipient cells. Cellular transport was the major functional role associated with membrane vesicles of diverse origins, both species and organelle-centric, however, recent decade has furnished unprecedented insights into the involvement of lipid vesicles in various biological phenomena ranging for cellular communication, bacterial and/or viral pathogenesis, aging, cancer progression and tumor invasion. In addition to these, their untapped clinical potential as diagnostic, therapenutic and prognostic modules is currently gaining attention (Wiklander et al., 2019). For up to date account of the MVs-based therapies in clinical research, readers are directed towards the review by Wiklander et al. (2019).

### What are the Various Kinds of MVs and How are They Produced?

#### Eukaryotic MVs

**FIGURE 1:**
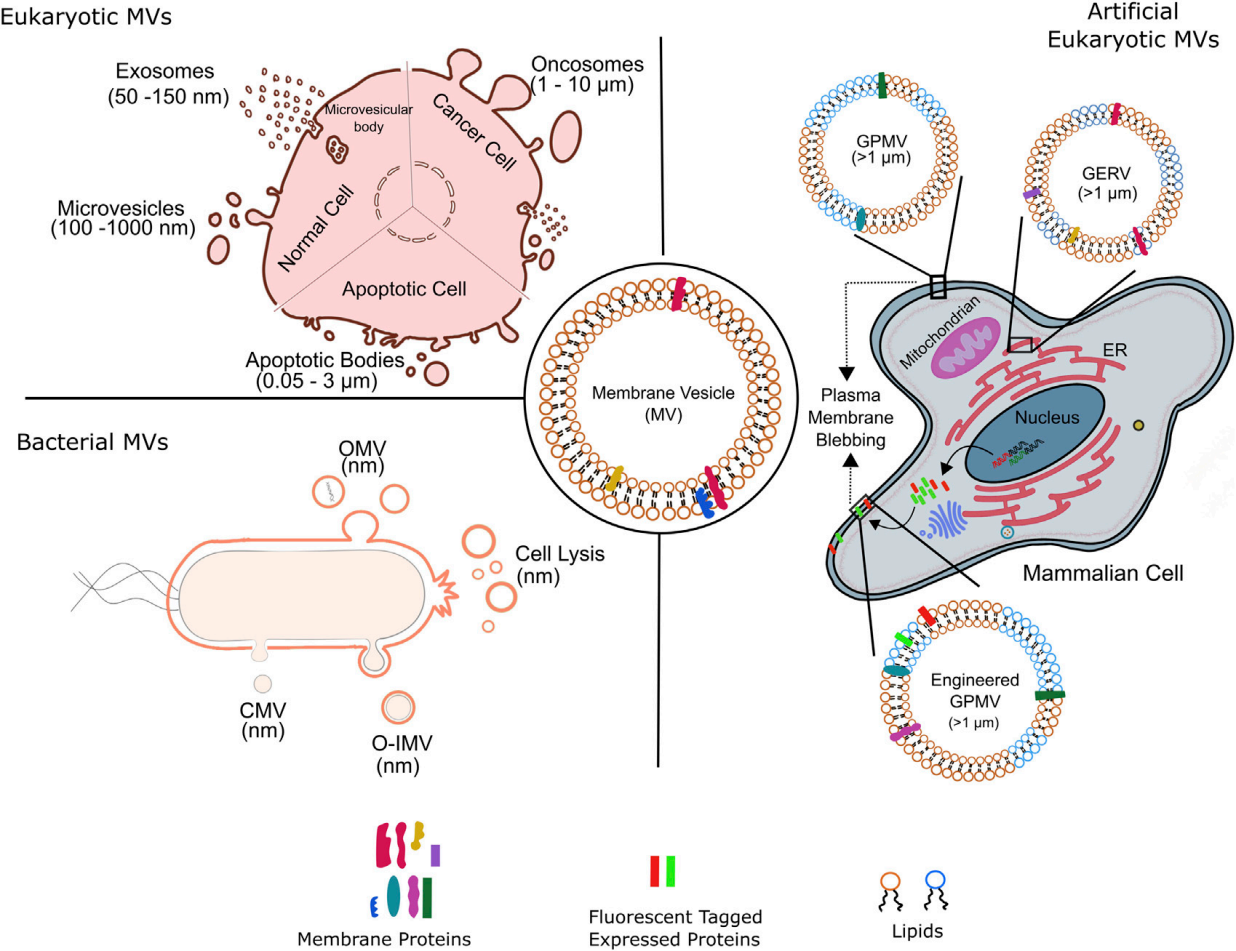
Membrane vesicles of variable sizes and origin. Schematic representation of various kinds of natural membrane vesicles from eukaryotic and bacterial cells. of the kinds of vesicles obtained from different cell types. Artificial membrane vesicles, giant plasma membrane vesicle (GPMV), synthesized from eukaryotic cells by chemical blebbing from plasma membranes of wild type or genetically modified cells. The exogenously expressed fluorescent tagged proteins in genetically modified cells are incorporated into the cell plasma membrane before GPMVs formation, forming the so-called engineered GPMVs. Giant Endoplasmic Reticulum Vesicles (GERs) represent membrane vesicles derived from endoplasmic reticulum (ER). OMV, outer membrane vesicle; CMV, cytoplasmic membrane vesicle; O-IMV, outer inner membrane vesicle; GPMV, giant plasma membrane vesicle and GERV, Giant endoplasmic reticulum vesicle.

MVs are diverse in origin and consequently known by various names. MVs of Eukaryotic origin including fungi and other parasites are microvesicles (100 nm–1 μm), exosomes (50–150 nm), oncosomes (1–10 nm), migrasomes (50–100 nm), apoptotic (0.05–3 μm) bodies and chemically induced giant plasma membrane vesicles (>1 μm) (Deatheragea and Cooksona, 2012; Todorova et al., 2017); the latter is associated with blebbing of plasma membrane (Figure 1). Eukaryotic microvesicles bud directly from the plasma membrane; contemporary exosomes are derived from the multivesicular bodies within the cell. Apoptotic bodies are produced by dying cells, oncosomes by large membrane protrusions primarily in malignant cells and migrasomes from amoeboid cells.

Microvesicles also known as micro particles are formed by outward budding from cell surface and share some properties with bacterial MVs. Though the exact details of the biogenesis of microvesicles is unknown, some studies highlight the connection between ESCRT pathway and early stages of microvesicle formation (Mayers and Audhya, 2012). Further, spontaneous membrane curvature to drive *de novo* microvesiculation has also been implicated, but largely rejected due to the fact that eukaryotic lipid membranes have active mechanisms to resist spontaneous curvature generation. Though, triggering of membrane curvature due to alterations in lipid and protein composition has not been ruled out. Particularly, uneven distribution of lipids between the two leaflets of lipid bilayer, modulated membrane biophysical properties such as rigidity and curvature leading to membrane budding. Moreover, specific membrane components responsible for microvesicles formation have been identified including cholesterol, Ras-related small GTPase, ARF6, and Rab proteins. Recently, a more generic model for *de novo* microvesicle formation involves protein crowding at the cell surface that generates pressure due to protein-protein and protein-lipid interactions driving membrane bending. Thus, enrichment of protein cargo at the site of microvesicle budding could be an efficient and simple mechanism to drive the formation of microvesicles.

Exosomes are formed from the outward budding of the late endosomal membrane leading to their accumulation in the multivesicular bodies (MVB) and the subsequent fusion of the MVBs with plasma membrane to release exosomes into extracellular space (Figure 1). ESCRT pathway in the first step of exosome biogenesis is well established; ESCRT independent mechanisms involving certain lipids, proteins and tetraspanins are also prevalent. The release of exosomes rendered by fusions of MVBs with plasma membrane is regulated by SNARE proteins, in addition to Rab and Ras GTPases; loss of function mutations in these proteins correlate with lesser exosome abundance.

Apoptotic bodies, like exosome are another type of MV specific to eukaryotes and are formed during apoptosis by outward budding from the cell surface (Figure 1); as a result, share some features with microvesicles. Unlike other MVs, the molecular cargo of apoptotic bodies consists of certain organelles and nuclear remnants. In addition to the main Eukaryotic MVs, cell-type specific MVs also exists such as oncosomes and migarsomes (Ma et al., 2015; Minciacchi et al., 2015). The various eukaryotic MVs mentioned above play roles of paramount importance in normal cellular functioning as well as disease progression. For instance, MVs are involved in tumor proliferation, invasion and evasion of immune system; cancerous cells produce higher amounts of MVs which are compositionally distinct than those produced by healthy counterparts. Eukaryotic MVs are also implicated in aging involving telomere regulation, as well as neurobiological diseases through transport of amyloid proteins. Finally, the use of MVs for clinical cancer diagnosis and monitoring of treatment response is gaining speed.

Apart from biological relevance, eukaryotic MVs also serve as molecularly complete scaffolds specific to plasma membrane and are used to investigate with intricate details the structure and function of native plasma membrane. These, giant plasma membrane vesicles (GPMVs), are on-demand MVs released from cells following chemically induced blebbing (Figure 1), and most likely involve similar steps as encountered during natural blebbing of plasma membrane during apoptosis or cell motility. Of note, actin contraction is not a perquisite for chemical induced GPMVs formation. The first chemically induced production of MV (i.e., GPMV) from mammalian cells was shown in isolated tumor cells and tissue (Belkin and Hardy, 1961) and this field has been expanding ever since. As outlined below, such artificial giant vesicles have proven highly useful as membrane models in a large number of biochemical and biophysical studies pertaining to revealing previously unknown insights to plasma membrane function, lipid domain dynamics and protein-lipid interactions with implications on protein sorting, lipid recruitment and cellular signaling.

#### Bacterial MVs

On the other hand, MVs from bacterial origin are known as outer membrane vesicles (OMV, μm) and outer-inner membrane vesicles (O-IMV, μm), Figure 1. Blebbing and subsequent pinching from the outer membrane surface produce qualitatively bacterial OMV; however, a general model and implicated mechanics and players remain to be elucidated. Reports suggests that increased vesiculation of outer bacterial membrane surface could be the major source of bacterial OMVs; primarily based on the discovery of bacterial mutants with hypo- or hypervesiculation phenotype. Almost 150 genes have been assigned to the process of vesiculation in Gram-negative *E. coli* (Kulp et al., 2015). Furthermore, mutations in the stress response pathway correlate to OMV biogenesis thus underlying the implication of OMV production in stress response. Weaker covalent linkages between the outer membrane and the underlying peptidoglycan layer resulting in the bulging and subsequently vesiculation of outer membrane have held the center stage as the predominant process of OMV production. This was supplemented by the involvement of misfolded proteins and peptidoglycan fragments that increase turgor pressure in the periplasm, leading to outer membrane bulging. Notably, some studies implicate OMV’s lipid constituent to drive their biogenesis by exploitation of charge-charge interactions. For example, distinct species of lipopolysacchride (LPS) harboring variable negative charges foster electrostatic repulsion leading to membrane protrusions and subsequently OMV generation. Additional membrane dependent aspects such as curvature have also been suggested however is limited to certain species such as *P. aeruginosa*. Finally, a more generic model of OMVs formation has emerged that focuses on the asymmetric phospholipid distribution in the outer and inner membranes and concomitant outward expansion of outer membrane to form OMVs under all growth conditions; invoking lipid composition dictated membrane curvature generation. In this regard, advances in lipidomics have greatly improved our knowledge on the lipid compositional differences between inner and outer bacterial membrane across various species. Of late, cell lysis has also emerged as a bonafide mechanism for OMV production. OMVs generated by grams-positive bacteria are also referred to as cytoplasmic membrane vesicles (Nagakubo et al., 2020). O-IMVs on the other hand are formed by the protrusion of both outer and cytoplasmic membranes through unknown mechanisms, though explosive cell lysis has been implicated (Toyofuku et al., 2019). In fact, how the O-IMVs transverses the complex bacterial cell wall is not known and a subject of immense investigations. The first evidence for existence of O-IMVs was demonstrated in *Shweanella vesiculosa* M7 and that the O-IMVs had entrapped DNA; O-IMV production was later found to be omnipresent across various bacterial species (Pérez-Cruz et al., 2013; Pérez-Cruz et al., 2015).

Bacterial MVs are indispensable mediators of intracellular communication due to transportation of various types of cargos. For instance, Gram-negative bacteria including *E. coli*, and *P. aeruginosa* use OMVs to transport virulence factors. OMVs containing virulence factors attach to the outer membrane leaflet of recipient cells and rapidly fuse allowing delivery of the cargo into the recipient periplasm. Apart from the widely studied gram-negative bacteria, gram-positive bacteria also produce OMVs enriched with surface associated virulence proteins that play a key role in the bacterial pathology. The vesicles also help in the transfer of proteins to other bacteria and eliminate intracellular competition. Acid-fast bacteria like *Mycobacterium tuberculosis* also release membrane vesicles that are packed with molecules that modulate the host immune response. *Mtb* OMVs usually contain virulent lipids such as lipoglycans and lipoarabinomannan, which inhibit phagosome maturation (Athman et al., 2015). Mtb OMVs are also shown to be produced upon trigger by environmental cues, like iron deficient environments, wherein such OMVs deliver iron and support proliferation of iron-deficient bacteria (Prados-Rosales et al., 2014b). In subsequent sections we detail the specific involvement of MVs in bacterial physiology, communication and virulence.

## Are MVs STRUCTURALLY AND COMPOSITIONALLY DISTINCT FROM DONOR CELLS?

Holistically, MV membrane consists of a phospholipid bilayer but can markedly differ from the donor cells in terms of lipid and protein composition. Recent advances in sophisticated mass spectroscopy based tools are enabling explicit insights into the lipidome and proteome of secreted MVs across various organisms (Skotland et al., 2017b; Ribeiro et al., 2018; Skotland et al., 2019), available in MV databases such as EVpedia (Kim et al., 2013; Kim et al., 2015) and Vesiclepedia (Kalra et al., 2012). Here we highlight the less explored lipid species enriched within MVs as lipids represent highly valuable markers for disease diagnosis.

### Lipidomic Analysis of Eukaryotic MVs

**FIGURE 2:**
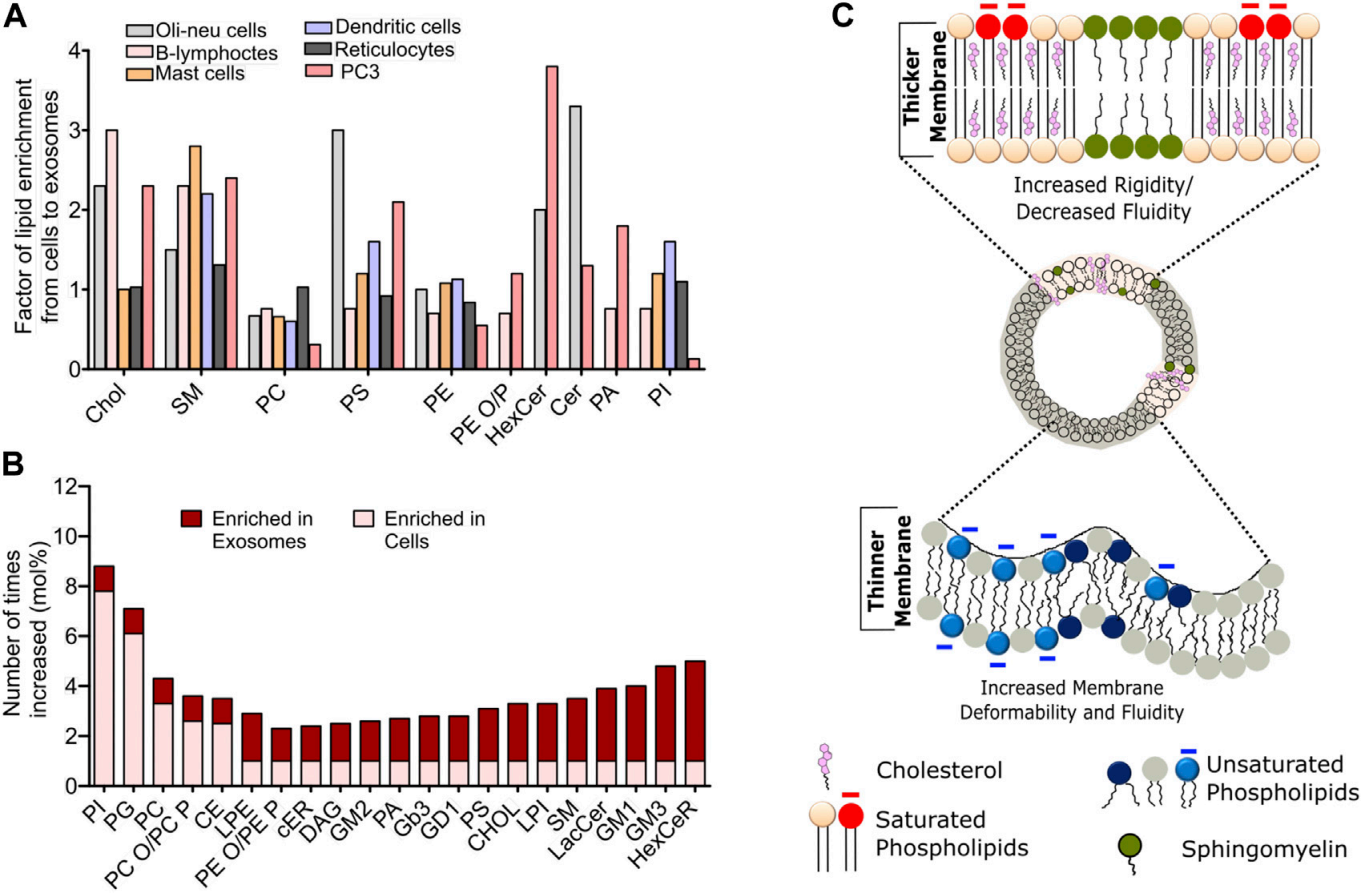
Compositional heterogeneity in membrane vesicles guide their membrane biophysical properties. **(A)** Fold change in the abundance of specific lipid species in exosomes compared to the donor cells across various cell lines. Data reprinted from Biochimica et Biophysica Acta (BBA) - Molecular and Cell Biology of Lipids, Llorente, A et al. Molecular lipidomics of exosomes released by PC-3 prostate cancer cells, 2013, 1831, 1,302–1,309. Copyright (2013), with permission from Elsevier **(B)** Relative changes in the abundance of indicated lipid constituents in the exosomes derived from PC3 cells. The data has been adapted with from Skotland et al. (2019) **(C)**. Schematic representation of biophysical attributes such as rigidity, membrane thickness and fluidity of cell-derived membrane vesicles dependent on the lipid composition. Enrichment of cholesterol and saturated lipid confer high rigidity and higher membrane thickness, while enrichment of unsaturated lipids leads to increased fluidity and higher membrane deformability.

Collective exosome lipidomic analysis from various groups across various cell types has revealed selective enrichment of certain lipid species (Figures 2A,B). For instance, the abundance of phosphatidylcholine (PC) and diacylglycerol (DAG) in exosomes is reduced compared to the membranes of their cells of origin, but are enriched in sphingomylein (SM), cholesterol (Chol), gangliosides (GS), and disaturated lipids in general; PE remains constant.

Studies on exosomes released from reticulocytes and PC3 cells show highest enrichment for Chol, SM, hexosylceramide (HexCer), lactosylceramide (LacCer) in PC3 secreted exosomes and moderate enrichment of SM in reticulocyte (Llorente et al., 2013). Comparison of lipid species from Oli-Neu cells, HEPG2 and PC3 cells demonstrate a cell-type specific effect on exosomal lipidome (Figures 2A,B). For instance, while all cell lines showed a similar lipid enrichment profile, Oli-Neu cells have highest enrichment of Cer and lowest of SM (Trajkovic et al., 2008). Based on extensive reviewing by Skotland et al. (2017b), it can be stated that Chol and SM represent the two major lipid species highly enrichment in secreted exosomes compared to their parent cells. An interesting study by Pienimacki-Roemer *et al.* analyzed lipidome of vesicles with varying sizes released from platelets and showed that Chol, SM, PI, and PS re common entities in all released vesicles types, but are selectively enriched in exosomes (Pienimaeki-Roemer et al., 2015). A recent work by Zang *et al.* demonstrated high levels of TAGS, DAGs, MAGs and cardiolipid in exosomes from AcPC-1. MBA-MB-231, and B16-F10 cells, in addition to PC and SM (Zhang et al., 2018). Furthermore, PS36:1, PS39:1, and PS37:0 was most abundant in exosomes from AsPC-1, while PS35:0 was the dominant species in other cell types. Notably, the presence of odd chained lipids was surprising, given the fact that such phospholipids are present in very scare amounts in cells. Exosomic lipid analysis of LIM125 colorectal cells identified 500 lipid species with high enrichment of TAG and CE (Lydic et al., 2015). Similar enrichment has been reported in the exosomes in U87 glioblastoma, Huh7 heptacellular carcinoma and human-bone marrow derived mesenchymal cell (Haraszti et al., 2016). Intriguingly, these studies showed high abundance of CL and not of SM in Huh7 and mesenchymal exosomes, with opposite trend for exosomes from U87 cell. Higher amounts of PC, PE, and PS with unsaturated fatty acid chain, e.g., 38:4 have been found in exosomes from platelets compared with mesenchymal stromal cells (Valkonen et al., 2019). Sun *et al.* characterized 264 lipids isolated from circulating MVs from human plasma and serum and revealed remarkably high abundance of lysoglycerophospholipids such as lyso-PC, lyso-PE, lysp-PI followed by SM and Cer (Sun et al., 2019). Exosomes released from the apical side of polarized murine cortical collecting duct principal cells has been shown to differ substantially from those secreted from the basolateral side, however further investigations are warranted to delineate the specific lipid changes. Lipid content of exosomes also shows remarkable differences depending on the tumourigenicity of the parent cell lines. For example, the seminal work by Brzozowski *et al.* showed glycerolipids and prenol lipids to be most abundant in exosomes from non-tumorigenic prostrate cells, while sterol lipids, SM and glycerosphingolipids were highly enriched in exosomes from highly tumorigenic and metastatic prostate cancer cells (Brzozowski et al., 2018).

MVs within biological fluids contain a heterogeneous population originating from various cell-types. For instance, MVs in seminal fluid-prostasomes are highly abundant in SM and HexCer while urinary exosomes are enriched with Chol. Further PS18:0/18:1 was found be second most abundant lipid after Chol in urinary exosomes and all major PE lipid types were identified as ether lipids. Recently Singhto *et al.* using MALDI-TOF-MS and TLC compared lipidome profile of urinary microvesicles and exosomes and revealed that mannosyl-di-PI ceramides were detected only in exosomes, whereas PI-ceramides were found in microvesicles (Singhto et al., 2019). Similarly, Chen *et al.* analyzed the MVs present in human serum fluid, and demonstrated high levels of TAG, CE 30:3 and PC 12:0, 1:0 ad 14:1 (Chen et al., 2019). Exosome harbors more phosphatidylserine (PS) compared to the plasma membrane in general, however conflicting reports exists regarding the extracellular or intracellular location of PS in the exosomal lipid bilayer.

### Lipidomic Analysis of Bacterial MVs

Compared to lipidome analysis of Eukaryotic MVs, there exist lesser number of reports on lipid analysis of bacterial MVs; though comparatively, Gram-positive bacteria are more investigated. Accumulation of phosphatidylglycerol (PG) and reduction of CL in MVs from *Streptococcus pyogenes* has been shown. Selective enrichment of cylindrical lipids has been proposed to foster MV formation in bacteria (Resch et al., 2016). MVs from *Propionibacetrium acnes* have reduced amount of TG compared to the parent cell membrane and may account for distinct membrane properties of MV membrane bilayers (Jeon et al., 2018). Compared to Gram-positive, Gram-negative bacteria have a distinct cell wall structure, and their MVs have been shown to be enriched in polar lipids such as PE, PG and diacylated phosphatidylinositol dimannoside (Ac2 PIM2); absence of mycolic acid ester implies the inner membrane to be the major site for MV origin (Prados-Rosales et al., 2011; Chowdhury and Jagannadham, 2013). MVs from *Mtb* under iron-deficient conditions are enriched with acylated glycerides and *P*, while under iron-sufficient conditions acyl trehalose was more abundant (Prados-Rosales et al., 2014b). Furthermore, higher abundance of saturated fatty acids in OMVs have been linked with low membrane fluidity of bacterial MVs.

### Does Modulated Lipid Profile Contribute to Altered Membrane Properties in MVs?

Membrane biophysical properties such as rigidity, elasticity, stiffness and order are crucial regulator of membrane fine structure, which in turn regulate various phenomena such as membrane curvature, lipid-protein interactions and their diffusion, cellular migration, adhesion and pathogen entry. Membrane properties in organelle specific fashion are critically controlled by the lipid compositional variation. For instance ER and Golgi have almost 10-fold lower amounts of SL and cholesterol with PM having maximum cholesterol content (Van Meer et al., 2008). This is the reason for high rigidity observed in plasma membrane compared to other intracellular membranes. Furthermore, lipid-specific structural variation impacts membrane curvature, which modulates membrane stiffness and elasticity. For example, phospholipid-like SM and phosphatidylcholine (PC) have cylindrical shapes due to their head and tail proportion. In contrast, lysophosphatidylcholine (LPC) and phosphoinositides (PI) have a higher head to tail ratio, leading to an inverted cone shape and a positive membrane curvature. On the other hand, PE, DAGs, TAGs, and PA have a cone-shaped structure due to their small head group, leading to negative membrane curvature. In line with these facts, a recently study on exosomes isolated from primary hepatocytes coupled with atomic force spectroscopy revealed these exosomes to be softer (less stiff) and less resistant to mechanical failure compared to those isolated from hepatocyte progenitor cell line MPL29 (Royo et al., 2019). This was attributed to the differential abundance of TAGs, PI, PE and LPC lipids between these exosomes. Study by Wubbolts *et al.* for the first time revealed high abundance of Chol in exosomal membranes with subtle similarities with detergent resistance fraction indicting that exosomes may have raft like membrane properties, namely, high order and rigidity (Wubbolts et al., 2003), Figure 2C. This fits well with recent reports showing exosomes to have high lipid order compared with microvesicles and apoptotic bodies released from same cells (Osteikoetxea et al., 2015). Moreover, exosome lipid bilayer due to the higher abundance of SM and di-saturated lipids has higher rigidity (Laulagnier et al., 2004), Figure 2C. This rigidity has shown to be pH dependent with low pH correlating to low rigidity and may be attributed to their site of origin, i.e., MVBs (Parolini et al., 2009). Moreover, the SM and Chol afforded high rigidity is also the reason behind degradation resistance behavior of MVs (Ridder et al., 2014). More work is needed to establish clear correlation with modulated lipidome and membrane biophysical properties and its impact on membrane-associated MV functions.

## What Are the Biological Functions of Cell-Derived MVs?

### Cellular Communication

**FIGURE 3:**
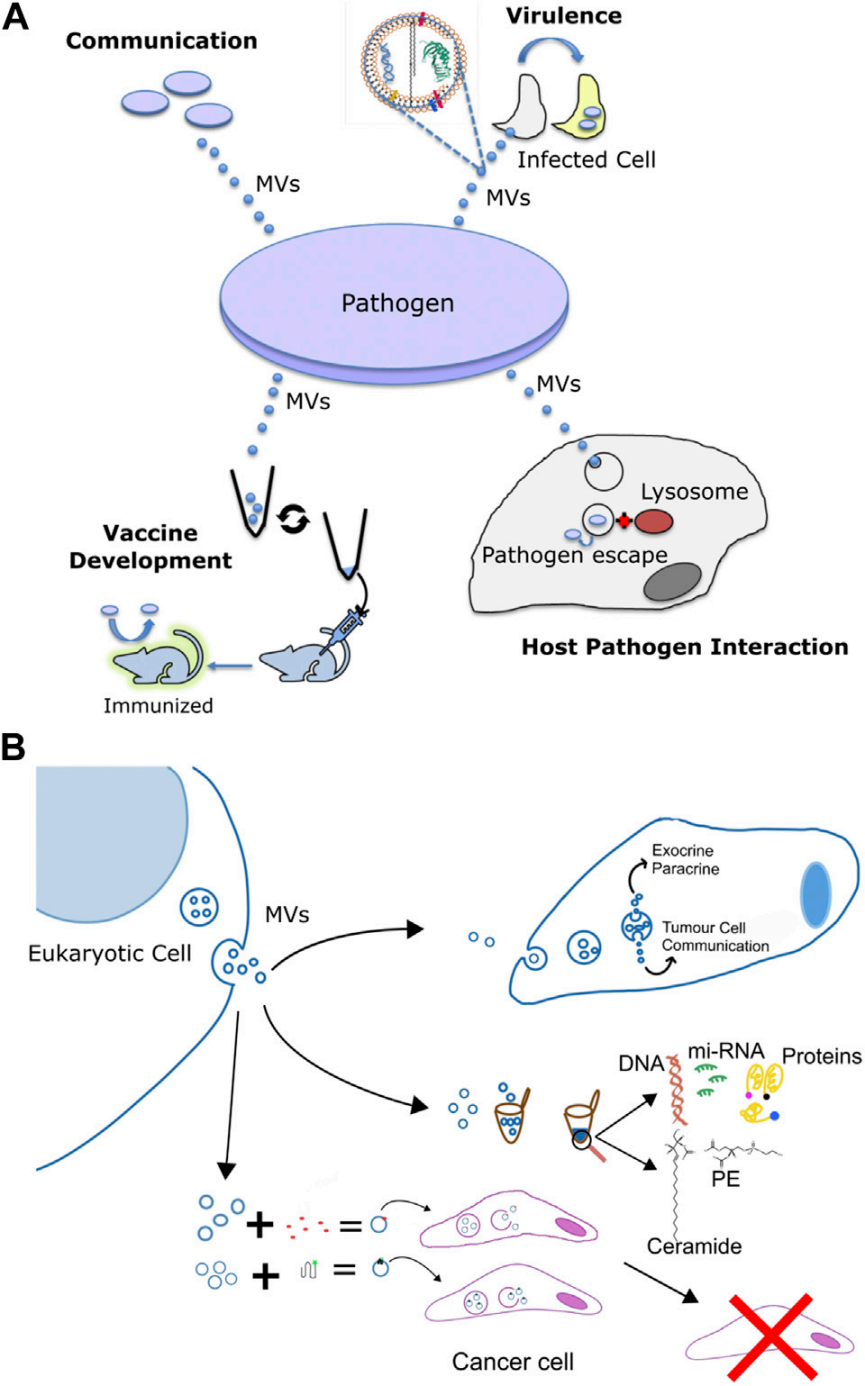
Multifaceted functions of membrane vesicles. **(A)** Pathogen derived membrane vesicles (MV) aid in communication, in the delivery of virulence factors to host cells during infection, as well as in altering interaction with the host and the use of the bacterial OMVs for the development of immunization against the pathogen. **(B)** Specific applications of eukaryotic membrane vesicles for 1) intracellular signalling and transport of chemicals, 2) to serve as minimally invasive diagnostic markers against various diseases by using the eukaryotic pathogen derived MV-enclosed molecular cargos such as DNA, proteins, lipids, and mi-RNA and 3) in recombinant cancer therapy via the combination of exosomes and nanotherapy for targeted cancer therapy. Exosomes derived from target cells are tagged with bioactive molecules (red) or functionalized protein (green) and injected into cancer cells or animal models for anti tumor therapy or help in immunization and vaccination against a particular cancer type.

Cell to cell communication or quorum sensing (QS) in bacterial species is the ability of individual cells to communicate with one another by the use of extracellular signaling molecules (Waters and Bassler, 2005). The most common signaling molecule produced by a large number of Gram-negative bacteria is L-homoserine lactones (AHLs). The N-acyl side chains of these molecules typically range from 4 to 18 carbons in length. The chain length imparts varying degrees of hydrophobicity and free diffusion is only possible for short chain molecules, while long chain molecules require transporters (Schaefer et al., 2002; Ying et al., 2007; Huang et al., 2012). Recent studies have shown the importance of MVs in the transfer of such signaling molecules. For instance, *Paracoccus denitrificans,* releases the hydrophobic C16-homoserine lactone (HSL) by fusion of HSL containing MVs with different bacteria; albeit with varying propensities (Toyofuku et al., 2017). This seminal study by Toyofuku *et al.* demonstrated a novel MV-based binary trafficking mechanism for the targeted and specific delivery of hydrophobic signaling moieties to other bacteria. MVs bound with low affinity to the well-known *P. aeruginosa* strain, however the freely diffusing non-MV C16-HSL, showed no major difference in the binding affinity to *E. coli* and *P. aeruginosa* strains thus underscoring the ability of MVs to recognize various cell types. Clearly MV aided transport of hydrophobic signaling molecules in open aqueous bacterial environment enables efficient cellular communication, as non MV associated signals would be highly diluted impeding sufficient accumulation required for QS. Another study using the marine pathogen *Vibrio harveyi*, also showed OMV-aided trafficking of hydrophobic QS molecule, CAI-1, a long-chain amino ketone, in aqueous environments (Brameyer et al., 2018) fostering communication with CAI-1 non producing *Vibrio cholerae* and *V. Harveyi*. Apart from AHLs, *P. aeruginosa* also secretes another QS molecule, 2-heptyl-3-hydroxy-4quinolone (*Pseudomonas* Quinolone Signal, PQS), which is another highly hydrophobic molecule, thus hampering its free aqueous diffusion. MVs are therefore used to traffic this molecule within a population (Mashburn-Warren et al., 2008), and the removal of these MVs has been shown to halt communication and inhibit group behavior under the PQS control (Mashburn and Whiteley, 2005). Thus, MVs play an important role in cell-to-cell communication by aiding in the trafficking of signaling molecules in a targeted manner (Figure 3A).

Along similar lines, MVs in eukaryotic cells also regulate cellular communication Particularly; their involvement in tempering with paracrine and autocrine communication in cancer is well studied (O’Brien et al., 2020), Figures 3A,B. Neoplastic cell release MVs, which modify the phenotype of recipient cells facilitating tumor growth (De Palma et al., 2017; O’Brien et al., 2020). For instance, ovarian cancer cells release microvesicles containing CD147 that promotes the expression of MMP-1,2- and nine- in endothelial cells (Yekula et al., 2020b; Lucchetti et al., 2020). Likewise, CD105 enriched microvesicles from renal cancer stem cells promote angiogenesis via stimulation of migration and tubule formation phenotype in endothelial cells (Parayath et al., 2020). Furthermore, angiogenic activity was stronger under hypoxic, oxygen deprived, conditions implying a regulatory role of tumor microenvironment on MV-mediated processes (Kumar and Deep, 2020). Similar to microvesicles, oncosomes also have been shown to participate in cell to cell communication and trigger migration of cancer associated fibroblasts (Minciacchi et al., 2017; Ciardiello et al., 2019). Notably, these fibroblasts in turn shed oncosomes with high content of microRNA-409 that contributes to EMT transition and cancer stem cell phenotype (Josson et al., 2015). Collectively, exchange of MVs, between cancer and normal cells regulate promotion of tumour growth, via not completely understood mechanisms.

### MV-Aided Release of Virulence Factors

Apart from being harbingers of communication signals, MVs also mediate transfer of cytotoxins, virulence factors and other biomolecules (Biller et al., 2014; Chattopadhyay and Jaganandham, 2015); many of these molecules lack identifiable signal sequences, Figure 3A. These MVs usually contain components of the bacterial outer membrane like proteins, lipopolysaccharides, phospholipids and peptidoglycans (Toyofuku et al., 2019; Nagakubo et al., 2020). For instance, *P. aeruginosa* use OMVs, to deliver an array of virulence factors such as *ß*-lactamase, alkaline phosphatase, hemolytic phospholipase C, and Cif, into the host airway epithelial cells via the fusion of OMVs with host cell lipid membrane. Particularly, host cell plasma membrane lipid rafts have been implicated as the most exploited site for fusion with bacterial OMVs carrying virulence cargo. N-WASP mediated actin pathway then traffics the vesicle cargo into the host cytoplasm (Bomberger et al., 2009) leading to systemic delivery to specific subcellular locations impact host processes. *Moraxella catarrhalis*, a pathogen responsible for the infection of the upper and lower respiratory tract (Augustyniak et al., 2018) use MVs to carry β-lactamase in high concentrations and subsequently helps protect susceptible strains of *M. catarrhalis* (Schaar et al., 2011b). Since *M. catarrhalis* is often found to be co-pathogens in infections with *S. pneumoniae* and *H. influenzae*, even these stains are protected against amoxicillin by the *ß*-lactamase rich OMVs by *M. catarrhalis* (Budhani and Struthers, 1998; Krishnamurthy et al., 2009). Interestingly, using bioinformatics and immunoproteomic approaches, Augustyniak *et al.* revealed various OMV associated proteins engaged in complement evasion and colonization strategies by *M. catarrhalis,* underlining their cross-reactive immunity (Augustyniak et al., 2018). Likewise, another proteomic study on the OMVs derived from clinically relevant *E. faecium* strains revealed the presence of proteins primarily associated with virulence factors, vaccine candidate and antimicrobial resistance, specifically vancomycin resistance (Wagner et al., 2018). *E. coli* and *Salmonella enterica* packs the cytotoxin ClyA with their OMVs that lyses the host cell membrane upon fusion. In fact, membrane lytic activity f ClyA was 8-fold higher when delivered via OMV pathway compared to its free form in solution. Similarly, targeted toxin delivery via OMV has been reported for *Bacillus anthracis* and enterotoxigenic *E. coli* as well. *H. pylori* also releases the vacuolating cytotoxin, VacA, associated with OMV for fusion with host cell membrane; VacA-containing MVs have less potent membrane lytic effect compared to ClyA. Mycobacterial MVs facilitate the release of various virulence-associated proteins and lipids during the course of infection. These include proteins such as LprG, LprA, LpqL, LppX, PBP-1, PSTS3 (Wang et al., 2019c) and lipids such as LAM, lipoarabinomannan, phosphatidylinositol, and acylated phosphatidylinositol-dimmannosides (Prados-Rosales et al., 2011) which have been isolated from intact bacteria or Mtb infected macrophage cells.

Similarly, eukaryotic microbes also release virulence factors packed with their MVs. Microvesicles ranging from 10 to 350 nm have been observed with various eukaryotic microbes such as *Leishmania*, *Histoplasma*, *Candida*, *Sporothrix*, and *Saccharomyces.* These are enriched in microbial lipids (including glucosylceramide and ergosterol), proteins and carbohydrates that are involved in virulence. These studies have collectively elucidated the functional involvement of MVs for release of virulence factors in close proximity of the host cell facilitating targeted delivery of pathogenic moieties not the target host cell. For example, proteomic analysis of the sectretome of *Leshmania donovani* revealed that 98% of the secreted proteins lack identifiable signal sequences required for secretion pathways, thus implicating non-classical MV (exosomes and microvesicles) mediated release during pathogenesis.

### MV-Regulated Host-Pathogen Infection Landscape

Apart from inducing an infection by targeted delivery of virulence factors, bacterial MVs also contribute to the complex host-pathogen interactions, specifically by tempering with the host immune response, Figure 3A. Typically, de-regulation of toll like receptor (TLR) and nucleotide-binding oligomerization domain containing protein (NOD) signaling has been extensively shown. For instance, *P. aeruginosa* OMVs activate TLR4 signaling pathway and contributes to the protection against pseudomonal lung disease in murine models (Zhao et al., 2013). Macrophage cells infected with *M. tuberculosis* or *M. bovis* release exosomes that contain pathogen-derived antigens including virulent lipid such as TDM, LAM etc. and activate host immune responses; both innate and acquired along with the production of pro-inflammatory cytokines (Schorey and Bhatnagar, 2008; De Toro et al., 2015). In fact lipoglycans such as LAM within *Mtb* MVs when released into infected macrophages provide a generic mechanism for these virulent lipoglycans to reach and inhibit T cells promoting immune evasion (Athman et al., 2017). *Moraxella* MVs induce pro-inflammatory response in epithelial cells with an upregulated IL-8 secretion resulting in the recruitment of TLR2 to the site of MV binding and ICAM-1 expression (Schaar et al., 2011a). MVs from *Salmonella typhimurium* potently stimulate APCs *in vitro*. They induce increased surface expression of MHC-II and CD86 and elevate the production of NO, TNF-α, and IL-12 to levels similar to that induced by whole bacterial cells (Alaniz et al., 2007). This shows the extent to which MVs can possess inflammatory properties and the ability to prime protective B and T cell responses *in vivo* against *Salmonella*. Similar observations on MVs from *H. pylori* leading to induction of IL-8 production in a MV dose dependent manner in a culture of gastric epithelial cells has been reported (Ismail et al., 2003). Typically, TLR and NOD ligands in bacterial MVs included capsular polysaccharide such as LPS, lipoproteins, peptidoglycan, and capsular proteins; supported by proteomic analysis. *Arabidosis* treated with MVs derived from *Xanthomonas* elicited an immune response in terms of defense gene activation, ROS burst and medium alkalinization (Bahar et al., 2016).

A variety of eukaryotic microbes, such as *Trichomonas vaginalis*, *Trypanosoma cruzi*, and helminths and intracellular parasites including *Plasmodium falciparum*, *Toxoplasma gondii* and *Leishmania spp*., have also been shown to employ their MVs for modulating host responses (Bhatnagar et al., 2007; Maxwell et al., 2008; Silverman et al., 2010; Martin-Jaular et al., 2011; Cestari et al., 2012; Marcilla et al., 2012; Mantel et al., 2013; Pope and Lässer, 2013; Regev-Rudzki et al., 2013; Twu et al., 2013; Marcilla et al., 2014; Arantes et al., 2016; Marti and Johnson, 2016; Schatz et al., 2017; Ribeiro et al., 2018). (Bhatnagar et al., 2007; Silverman et al., 2010; Martin-Jaular et al., 2011; Cestari et al., 2012; Marcilla et al., 2012; Mantel et al., 2013; Pope and Lässer, 2013; Regev-Rudzki et al., 2013; Twu et al., 2013; Marcilla et al., 2014; Arantes et al., 2016; Marti and Johnson 2016; Schatz et al., 2017; Ribeiro et al., 2018). For example, *Leishmania spp*. constitutively secrete exosomes within the lumen of the sand fly midgut and, following their ingestion into a new host these MVs play an important role in modulating host immunity promoting parasitic infection (Athman et al., 2015). In *T. cruzi*, a difference in MV production between strains has been shown to correlate with their infectivity and virulence affecting the host-parasite interactions, particularly invasion and proliferation (Ribeiro et al., 2018). Collectedly, MVs not only play a role in establishing an infection but also in the immune response associated with the disease.

## Metabolic Reprograming Induced by MVs: FUNCTIONAL IMPACT ON CANCER PROGRESSION AND DRUG RESISTANCE

Here, we shed light on the recent findings to show how MVs play a crucial role in reprogramming of cancer metabolism that in turn modulates cancer progression, metastasis, and drug resistance.

### Deregulated Metabolism in Cancer Cells

Cancerous cells often encounter metabolic stresses due to their rapid growth which are characterized with deprivation of nutrients such as glucose, glutamine, lipids and oxygen within tumour microenvironment. However, the cancer cells adapt to such deficiencies by altering its metabolic pathways. For instance, under glucose deprivation, cancer cells upregulate the energetically and metabolically wasteful erobic glycolysis (Hay, 2016). In other cases, increased uptake of glutamine and amino acids is documented (Altman et al., 2016). Several genetic and epigenetic alterations endow cancer cells to manoeuvre alternate strategies for the production of all the necessary metabolites required for survival and proliferation. For instance, various receptor-mediated signalling pathways, such as Ras, RAF–mitogen-activated protein kinase (MAP kinase), phosphatidylinositol 3-kinases (PI3Ks) and mammalian target of rapamycin (mTOR) are exploited simultaneously. Alteration of these, rendered by activated oncogenes c-Myc (Myc), nuclear factor kB (NF-kB), AKT, and the tyrosine kinase receptors (epidermal growth factor, EGF; insulin-like growth factor 1, IGF-1; Her-2; etc.) foster glucose uptake by upregulating the expression of the glucose transporter GLUT1 and glycolysis (Levine and Puzio-Kuter, 2010). In addition to enhanced glycolysis, Myc also upregulates glutaminolysis by stimulating the transcription of glutaminase-1. This supports fatty acid (FA) synthesis, promotes mitochondrial gene expression and biogenesis (De Berardinis and Chandel, 2016). PI3K/AKT-mediated suppression of FA oxidation contributes to enhanced lipogenesis in proliferating cells. In addition, lipogenic genes including acetyl-CoA carboxylase (ACACA); FA synthase (FASN) and stearoyl-CoA desaturase (SCD) are induced by the mTORC1 activated sterol-regulatory element-binding protein family, which subsequently promote increased fatty acid biosynthesis (De Berardinis and Chandel, 2016). Another oncogene, KRas, under nutrient stressed condition, scavenges extracellular proteins to produce glutamine and other amino acids to fuel the tricarboxylic acid (TCA) cycle for survival and growth in pancreatic cancer (De Berardinis and Chandel, 2016). KRas also reprograms glucose metabolism by upregulating glycolytic flux via modulation of GLUT1, HK1 (hexokinase1), HK2 (hexokinase2), PFK1 (phosphofructokinase 1) and LDHA (lactate dehydrogenase A) and thus fulfill the increasing energy demands and biosynthesis (Bryant et al., 2014). Of note, some common oncogenes, such as chromatin binding protein polybromo 1 (PBRM1), which are otherwise not directly linked to above pathways, also play a profound role in cancer metabolism to support metabolic rewiring (Slaughter et al., 2018). Aside from oncogenes, tumour suppressor such as p53 transcription factor plays a pivotal role in metabolic stress management. p53 promotes a transcription-independent up-regulation of pentose phosphate pathway (PPP), which is essential for the production of lipid and nucleotide biosynthesis. p53 transcriptional target, TIGAR (TP53-induced glycolysis and apoptosis regulator), contributes to oxidative PPP up-regulation (Reid and Kong, 2013). Furthermore, p53 also maximizes mitochondrial oxidative phosphorylation (OXPHOS) by inducing the expression of ETC (electron transport chain) assembly factor *SCO2* (Reid and Kong, 2013). In response to low glucose levels, another tumour suppressor AMPK (AMP – activated protein kinase), phosphorylates and activates p53. Activated p53 then enables adaptation to deprived glucose condition through metabolic check point arrest that provide rest to the cells along with a prolonged wait time in pro-proliferative conditions (Reid and Kong, 2013).

### How do MVs Rewire Cell Metabolism to Modulate Cancer Progression?

Proteomics studies on eukaryotic MVs from cancer cells (i.e., exosomes) have elucidated them to be enriched with GLUT1 and glycolytic enzyme PKM2. For instance, Wan et al. showed enriched PKM2, and GLUT1 levels in secreted exosomes from hepatic stellate cells promoting metastasis, while another study demonstrated increased PKM2 exosomal content to be associated with pre-metastatic niche (Dai et al., 2019; Wan et al., 2019). Importantly, inhibition of exosome secretion by GW4869 inhibits glycolysis and reverse the phenotype restricting cancer progression, illustrating the potential of targeting exosomal pathways for anti-cancer therapy. Similarly, increased exosomal VEGF is associated with enhanced glycolysis in HUVECS (Wang et al., 2019a). Very recently, Sung et al. have demonstrated that triple negative breast cancer derived exosomes transport ITGB4 proteins that induce mitophagy, promote MCT4 expression in recpient cells that in turn increases glycolysis and lactate transport (Sung et al., 2020). In addition to proteins, exosomal cargo such as microRNA are also implicated in cancer cell metabolic reprgramming. For example, breast cancer cell derived exosomal miR-111 supresses glucosie uptake by niche cels by downregulating PKM2 and GLUT1; inhibition of miR-122 rescues glucose metabolism and restricts metastasis (Fong et al., 2015). Another breast cancer derived exosomal miR, miR-155 promotes lipolysis in adipocytes by decreasing PPARy expression (peroxisome proliferators-activated receptor (Wu et al., 2018). Recently, Shu et al. showed that human melanoma cells derived exosomes enriched with miR-155 and miR-210 remodel stromal cell metabolism by enahcing glycolysis and supressing OXPHOS expression (La Shu et al., 2018) which facilitates tumor migration and invasion. The same authors also interestingly showed that exosomes transferred with respective miRNA inhibitors can reverse the exosome-mediated metabolic reprogramming, and hence reduce the risk of tumor metastasis. FASN has been identified in prostate cancer cell derived exosomes hinting at their role in cancer cell lipogenesis (Lázaro-Ibáñez et al., 2017). Apart from the FA pathway, sugar related pathways are also modulated by MVs. For instance, proteomics of HCC showed a enhanced presence of enzymes associated with glycolysis (Zhang et al., 2017). In fact, glycolytic enzymes are some of the most commonly identified proteins in EV based proteomics and is accordance with the high levels of ATP in the tumor environment (Arab and Hadjati, 2019) which can help in the enhanced levels of adenosine and hence help with the immune escape of cancer cells (Vigano et al., 2019).

### How do MVs Contribute Toward the Development of Chemo and Radioresistance?

One of the most important treatment methods for cancer that is administered either on its own or along with chemotherapy is radiotherapy. As with any treatment for cancer, one of the major obstacles is the recurrence and relapse of the tumor and its resistance to radiotherapy (radioresistance) (Szatmári et al., 2019). Owing to the fact that majority of people do receive radiotherapy, it is a key issue that has been greatly studied to understand the underlying mechanism and cause of radioresistance. During radiation therapy, there is direct damage due to the energy of the radiation or indirect effects due to the reactive oxygen species on the target cells. However, not all cells are under the direct effect of the radiation and are prone to bystander effects. These bystander cells receive signals from damaged cells via the help of MVs (Ni et al., 2019).

Glioblastoma (GBM) based radiation therapy has been shown to possess limited efficacy due to the enrichment of cancer stem cells (CSCs). CSCs have the capacity to cause recurrent rumors and are resistant to therapies usually used against GBMs (Yekula et al., 2020a). In this regard, a study by Ramakrishna et al., showed that mass exporting of miR-603 in MVs led to the treatment resistant phenotype. The decreased levels of miR-603 drove the post radiation therapeutic resistance of GBM leading to the formation of IGF-1 mediated stem cell state in the GBMs. It was seen that ectopic expression of the miR-603 reversed this effect contributing to a positive effect of radiation and tumoricidal agents (Ramakrishnan et al., 2020). Studies of head and neck cancer cells have shown MVs to contain chromatin fragments having a functional significance toward signaling between irradiated and non-irradiated cells. Apart from this, altered levels of exosome markers were observed; while CD63 and CD81 levels were increased, CD9 and TSG101 levels remained unchanged. A similar upregulation of HSPs in exosomes released by the irradiated cells (Abramowicz et al., 2019) has also been documented. A study by Mrowczynski et al. demonstrated the effect of irradiated cells derived exosomes on recipient cells *in vitro* and *in vivo* models of nervous system cancer. The provided evidence that these exosomes contain upregulated oncogenic and downregulated tumor suppressive contents. There were downregulated levels of tumor suppressive miRNA like miR-365 and miR-516 which increased cancer cell proliferation and upregulated level of miR-889 that increased resistance, metastasis and decreased apoptosis. These collectively show that radiation exposure leads to the release of altered exosomes that contain signals which decrease tumor suppressive cargo and concomitantly increase oncogenic cargo to be taken up by the recipient cell leading to proliferation and enhanced ability to survive radiation treatment (Mrowczynski et al., 2018).

Similar to radioresistance, cancer cells also relay cargo and other components that are important for survival of neighboring cells in response to stress conditions brought about by chemotherapeutic drugs. For example, multiple myeloma (MM), a plasma cell malignancy has a range of immunomodulatory drugs, proteasome inhibitors and antibody drugs developed against it. MM cells are seen to acquire resistance on long term exposure to these drugs. MM derived exosomes have been shown to play a role in the transmission of this information from cell to cell and subsequently help in the cancer progression and drug resistance of surrounding cells (Colombo et al., 2019; Mogollón et al., 2019). Exosomes from breast cancer cells (MDAMB231) treated with paclitacel (PTX) has been shown to contain a cell survival protein and cancer marker, Survivin. These Survivin enriched exosomes promoted cell survival of fibroblasts and SKBR3 breast cancer cells when challenged with PTX treatment. Thus, these altered exosomes play an important role in mediating resistance to PTX (Kreger et al., 2016). Similar observations have been made with respect to resistance of breast cancer cells to Doxorubicin (DOX). For instance, the proteome profile of MCF-7/ADR cells show increased expression of CD44 in the DOX-resistant cells compared to that of the parental cell line. This leads to a increased exosome-mediated intercellular transfer of proteins in the cancer cells and its chemoresistance regulation. A siRNA based approach to target the CD44 could efficiently silence the expression of CD44 and in turn enhance the susceptibility of the cells to DOX and hence reduce cell proliferation (Wang et al., 2020b). A recent study by Faict et al. revealed that exosomes enriched with high acid SMase (ASM) content can transfer drug-resistant phenotype to drug-sensitive cells by altering lipid metabolism (Faict et al., 2019). Exosomes from patients with Kras chemoresistant lung cancer patients have been shown to remodel metabolism in a PKM2-dependent manner to maintain lung cancer cell metabolic chemoresistance (Petanidis et al., 2020). All these studies point to the MVs-mediated therapy resistance generation in cancer and consequently the potential of targeting exosomes in tumor drug resistant therapy.

## Can MVs SERVE AS BONAFIDE VACCINE CANDIDATES?

Recently the ability of MVs from various pathogens to act as antigen decoys modulating antibody response has been reported. MVs released by various pathogenic organisms are nonviable, however are enriched with antigens that are recognized by the host adaptive immune system (Figure 3A). Moreover, studies focused on characterization of MV released *in vivo* during infection, and from sera of patients suffering from bacterial infections further reinforce the conclusion that MVs represent an excellent source of antigens during infection capable for eliciting a bonafide immune response. This aptly lays the foundation of exploring MVs in vaccine campaigns, in addition to serving as potential biomarkers as discussed in subsequent sections.

Due to the intrinsic adjuvant capability of bacterial MVs, they have been successfully applied in various vaccine platforms. One of the most promising OMV vaccines till date is the MenB OMV vaccine for Meningitis type B (MenB), caused by *Neisseria meningitides* (Nm) (Petousis-Harris, 2018). The foremost rationale for its development was the limited efficacy of the traditional vaccine formulation against Nm serogroup B attributed to the risk of autoimmune response due to the homology of the Nm polysaccharide antigen with fetal neural tissue. Thus, group B strain specific vaccine was derived from Nm OMV expressing the immunodominant protein Porin A. As of now, two OMV-containing group B vaccine are available, a detergent extracted OMV (D-OMV), Bexsero (Novartis) and VA-MENGOC-BC (Giuliani et al., 2006; Serruto et al., 2012; Petousis-Harris, 2018). Bexsero is combined with highly immunogenic antigens–fHbp, NadA and NHBA, of the MenB resulting in a formulation fit for human use. Interestingly, VA-MENGOC-BC has been shown to confer immunity against gonorrhea as well, underlying the cross-species utility of cell-derived MVs. Recently, OMVs from *H. pylori* have also been demonstrated as efficient adjuvants against *H. pylori* infection by producing a robust Th1/Th2/TH17 immune responses, and are more effective compared to the cholera toxin in all vaccine types resulting in better cell-mediated and humoral immunity (Song et al., 2020). In another study, Codemo *et al.* demonstrated the potential of OMVs from *Streptococcus pneumoniae* in attenuating pneumococcal evasion in lung epithelial and human monocyte derived dendritic cells by inducing proinflammatory cytokine response (Codemo et al., 2018). Similarly, OMVs derived from *Vibrio cholerae* shows long lasting immunity (Schild et al., 2008), and those from *Mtb* are effective as live BCG immunization in protecting against *Mtb* infection (Prados-Rosales et al., 2014a). Furthermore, mice immunized with OMVs from *P. aeruginosa* have been shown to possess protection against subsequent lethal infections; due to efficient stimulation of murine inflammatory factors via the TLR4 signaling pathway. This could provide protection against pseudomonal lung disease in murine models (Zhao et al., 2013). PNAG surface polysaccharide (poly-N-acetyl-D-glucosamine) is produced by a broad range of bacteria, fungi and protozoan cells. Vaccination based on this surface antigen could reduce the disease caused by such microbes. A glycosylated outer membrane vesicle (glyOMVs) released from laboratory strains of *E. coli* expressing the PNAG surface polysaccharide proved to be effective in imparting protective immunity in mice against two distinct PNAG positive bacterial species–*S. aureus* and *F. tularensis* subspecies *holarctica* (Stevenson et al., 2018). Engineered OMVs expressing five *S. aureus* protective antigens released from *E. coli* were shown to elicit high, saturating antigen-specific antibody titers in mice which imparted a protective immunity in them against *S. aureus* Newman strain (Irene et al., 2019). Likewise, exosomes released from mycobacteria-infected macrophages have been shown to generate memory T cells in mice indicating their potential utility as an *M. tuberculosis* vaccine (Cheng and Schorey, 2013). In continuation, natural and artificial MVs released by various strains of mycobacteria have demonstrated protective immunogenic effects in various animal models (Prados-Rosales et al., 2014a). Additional research is required to propel clinical use of Mtb based EVs as vaccines. On a different note, a recent study by Kim *et al.* showcased immunotherapeutic properties of bacterial OMVs rather than as vaccine candidates. Their results revealed long-term antitumor immune responses by bac OMVs which could fully eradicate established tumors by induction of antitumor cytokines CXCL10 and interferon-γ (Kim et al., 2017).

Similar to bacterial MVs, Eukaryotic MVs have also been shown to possess immunomodulatory properties and serve as excellent candidates as adjuvants in various anti-fungal vaccine developments (Freitas et al., 2019; Brauer et al., 2020). An important study by Nogueira *et al.* elucidated a non-overlapping immunomodulatory response from MVs derived from three distinct *Leishmania* species that cause different clinical manifestations (Nogueira et al., 2020). This study highlights the potential of developing strain specific eukaryotic MV-based vaccine adjuvants against *Leishmania* infections.

Notably, some serious concerns in the commercial/clinical application of bacterial OMVs in specific and MVs in general as vaccine adjuvants remains. First is the toxicity of LPS inherently found in all major bacterial OMVs, which needs to be optimized. This could be achieved by the removal of OMV endotoxins post OMV production. This thus highlights the need to investigate optimal MV design to balance toxicity and immunogenicity. Further, mass production of MV for use in humans is another deterrent and calls to attention investigations centered on optimizing and streamlining a consistent process of MV production with less batch variability. In addition, the antigen-loading efficiency of exosomes should also be improved in future.

## What Are the Theranostic Avenues for MVs?

### Cancer Biomarkers

Microvesicles and exosomes are the two major kinds of MVs involved in directional exchange of molecular messengers between cancer cells. Particularly, circulating exosomes found in various of samples, such as urine, blood, bile, etc. harbor tumor-specific molecular signatures including oncoproteins, nucleic acids, lipids and miRNAs and hence demonstrate features as next-generation non-invasive biomarkers for liquid biopsy in cancer diagnosis (Figure 3B) (Table 1). In addition, the ability to naturally surmount biological barriers makes them attractive modules for exploitation in cancer diagnosis (Morad et al., 2019).

**TABLE 1:** Membrane Vesicle Based Biomarkers in various cancer types.

Condition	Biomarker assessed	Source	
Pancreatic cancer	GPC-1	blood	Melo et al. (2015); Fais et al. (2016)
Glioma	EGFR *NLGN3* and *PTTG1* mRNA	Serum	Wang et al. (2019b)
Breast cancer	Phosphoproteins	Plasma	Chen et al. (2017)
Liver diseases	Platelet factor 4	Serum	Nguyen et al. (2019)
Pancreatic and Pancreatic ductal adenocarcinoma	Kras DNA mutants	blood	Allenson et al. (2017); Yang et al. (2017b)
Lung adenocarcinoma and Granulomas	miRNAs	plasma	Cazzoli et al. (2013)
Breast cancer	miR-21	serum	Gao et al. (2019); Zhao et al. (2020)
	miR-375		
Colorectal cancer	miR-19a	serum	Matsumura et al. (2015)
Prostate cancer	CH_2_ stretching in lipids and proteins via IR	blood	Lee et al. (2018)
Prostate Cancer	Levels of various lipid species	Urine	Skotland et al. (2017a)
	Ceramide species	Urine	Clos-Garcia et al. (2018)
Pancreatic Cancer	LPC (22:0); PC(14:0/22:2)	Serum	Tao et al. (2019)
	PE (16:0/18:0)		
Multiple Sclerosis	Glycosphingolipids and sulphated galactosylceramide	Plasma	Moyano et al. (2016)

The protein and nucleic acid contents of MVs have been thoroughly evaluated as biomarkers for disease diagnosis and prognosis (Fais et al., 2016). For instance, exosomal levels of glypican 1 (GPC-1), proteoglycan, in the blood was assessed for detection of pancreatic cancer. GPC-1 positive exosomes enabled distinguishing healthy individuals from those with early and late stage pancreatic cancer and from those with benign pancreatic disease (Melo et al., 2015). In fact, GPC-1 and its regulatory miRNAs have already been reported as specific biomarkers for the diagnosis of colorectal cancer as well (Melo et al., 2015). In another study protein expression of EGFR^+^ EVs were illustrated as effective diagnostic and prognostic markers of glioma, wherein EGFR expression in serum EVs were able to accurately differentiate high-grade and low-grade glioma patients (Wang et al., 2019b). The authors also showed the potential of *NLGN3* and *PTTG1* mRNA in EVs for detecting glioma patients. Interestingly, protein phosphorylation status is an emergent marker for early stage cancer. Chen et al. demonstrated that patients with breast cancer exhibit significantly higher levels of phosphoproteins in plasma MVs than healthy controls (Chen et al., 2017). Platelet factor 4 protein has surfaced as a new exosomal biomarker in the serum of patients with different liver diseases (Nguyen et al., 2019).

Exosomes containing mutants of Kras DNA have been used for predicting the status of pancreatic cancer and pancreatic ductal adenocarcinoma (Allenson et al., 2017; Yang et al., 2017b). Multiple exosomal miRNAs also have been used for distinguishing patients with lung adenocarcinoma and granulomas (Cazzoli et al., 2013); microRNAs (miRNAs) are a class of small, noncoding RNAs. Exosomal miR-375 expression level can accurately differentiate estrogen receptor-(ER) positive breast cancer at early stages (stages I, II) with a high accuracy (Zhao et al., 2020). Likewise, miR-21 has been used to differentiate breast cancer patients from healthy counterparts (Gao et al., 2019; Zhao et al., 2020). Furthermore, expression of exosomal *miR-19a* in serum has been identified as a prognostic biomarker for recurrence in CRC patients (Matsumura et al., 2015). In a recent study, Lee et al*.* employing a label free Raman MV fingerprinting approach identified diagnostic MV signatures such as CH_2_ deformation stretching in lipids, proteins, and C=C stretching in lipids and were capable of distinguishing prostate cancer patients compared to healthy donors (Lee et al., 2018) adding to MV based diagnostic tool kit in prostate cancer.

The use of exosomal lipids as cancer biomarkers is not very widespread and currently emerging due to advances in lipidomics and effective methods for lipid isolation. In a seminal study by Skotland et al., analysis of exosomal lipids from the urine of prostate cancer patients revealed significant enrichment of LacCer 18:1/16:0 compared to control healthy controls. In contrast, PS 18:1/18:1 was highly abundant on control healthy samples. This method demonstrated a high sensitivity (93%) and specificity (100%), however reinforced the application of MS based lipidomic platforms as a routine method for clinical practice. This was due to the fact that various lipid classes displayed very little differences, which would go undetected using other semi-quantitative approaches (Skotland et al., 2017a). Specific to prostate cancer, several other Cer species have been found to be significantly different between stage 3 and 2 cancer patients (Clos-Garcia et al., 2018). Other work has furnished lipid species such as TAG and CE expressed in high amounts in urinary exosomes from prostate cancer patients (Yang et al., 2017a), however caution should be exercised when dealing with these lipid classes as these can be fall out of ineffective exosomes isolation causing contamination of TAG and CE enriched lipid droplets. Significantly deregulated lipid species, including LPC (22:0), PC (P-14:0/22:2), and PE (16:0/18:1), in serum exosomes, have been shown to be associated with tumor stage and tumor diameter in pancreatic cancer patients (Tao et al., 2019). In another very recent work, lipidomic analysis of urinary exosomes in α-tryptasemia patients elucidated 64 out of 521 lipid species from 19 lipid classes to be significantly different compared to healthy controls (Glover et al., 2019).

Apart from urine, other bio fluid MVs has also furnished lipid profiles amenable for diagnostic purposes. For example, serum EVs from 20 pancreatic cancer patients and healthy controls revealed PE 16:0/18:1 to be associated with the tumor stage, and significantly correlated with patient overall survival (Tao et al., 2019). Another study reported lipidomic analysis of MVs from plasma of multiple sclerosis patients and healthy volunteers and revealed presence of sulfatides, a class of glycosphingolipids; sulfated galactosylceramide. Particularly sulfatide C16:0 was elevated in the EVs isolated from patient samples (Moyano et al., 2016). Finally, Hough *et al.* isolated EVs from bronchoalveolar lavage fluid of asthmatics and healthy controls, and reported SM 34:to be highly abundant in EVs from second-hand smoke exposure asthmatics compared to healthy controls (Hough et al., 2018). Thus, lipid from various bio fluid MVs can serve as robust biomarkers for cancer diagnosis and prognosis, however this field has to reach its full potential in years to come.

### Tuberculosis Biomarker

Incorporation of mycobacterial proteins in exosomes released from infected macrophages has been exploited to elucidate robust and high fidelity biomarkers for *tuberculosis* (Kruh-Garcia et al., 2015). For example, an interesting study was able to differentiate between pulmonary and extra pulmonary *tuberculosis* based on the peptide markers (corresponding to Antigens 85b, BfrB, GlcB, Mpt64, and HspX) found in the serum exosomes of patients suffering from these, compared to healthy controls. Interestingly, some of the healthy patients (absence of active TB) showed evidence of latent disease dependent on the abundance of specific biomarker mycobacterial peptides belonging to (Kruh-Garcia et al., 2014). OMVs from BCG or *Mtb* have been demonstrated to elicit a cellular response while conferring protection against an *Mtb* aerosol (Prados-Rosales et al., 2014a). Moreover, *Mtb* MVs has also been shown to boost the effect of the BCG vaccine; surfacing as effective adjuvants. Probably, *Mtb* MVs may serve as vaccine even in the absence of adjuvants, due to the lipid immunogenic components such as TDM, LAM etc. However, the full diagnostic, prognostic and therapeutic value of MVs with respect to tuberculosis has yet to be fully realized.

### For Therapeutic Delivery and Tissue Regeneration

The earliest report on the use of exosomes for drug delivery was reported by Zhang et al., wherein an enhanced anti-inflammatory effect by exosomal delivery of curcumin to target inflammatory cells was shown. Since then exosomes have been intensively investigated to encapsulate various therapeutic drugs for disease treatment (Sun et al., 2010). For instance, exosomes loaded with doxorubicin (DOX) and imperialine exhibit excellent potential for cancer treatments (Fan et al., 2019; Lin et al., 2019). Wu *et al.* developed engineered exosomes by coupling it with nanoparticles functionalized with platinum Pt (IV) prodrug (Pt (lau)), human serum albumin (HSA), and lecithin (Xiong et al., 2019). When compared with free cisplatin, the engineered exosomes facilitated breast-cancer-targeted drug delivery due to the tumour-homing ability of the macrophage-secreted exosomes. In another example of engineered MVs, Bi_2_Se_3_ nanodots and DOX co-embedded microparticles (Bi_2_Se_3_/DOX@MPs) were fabricated. Using ultraviolet light irradiation-induced budding of parent cells preloaded with Bi_2_Se_3_ nanodots and DOX, the drug-loaded microvesicles displayed dual-modal imaging capacity and excellent tumor suppression (Wang et al., 2020a). In a seminal work, using cancer-derived exosomes to deliver palladium catalyst in cells, a localized prodrug activation could be achieved; this underlines the development of new targeted therapy module-exosome-directed catalyst prodrug therapy (Sancho-Albero et al., 2019). In the last two years, indirect loading of drugs and targeting-ligands to produce functionalized MVs *in vivo* for drug delivery have surfaced (Tang et al., 2019; Yong et al., 2019; Zhang et al., 2019). Exosomes derived from immature dendritic cells lacking immunostimulatory markers like CD40, MHC-I, MHC-II were produced and engineered to target tumors by expressing lysosome associated membrane glycoprotein 2b (Lamp2b) which was fused with iRGD targeting peptide for αv integrin. These iRGD exosomes were then loaded with doxorubicin. These iRGD-exosomes showed high affinity to αv integrin positive breast cancer cells both *in vitro* and on BALC/c nude mice *in vivo*. This strategy helped in the administration of a low dose of doxorubicin that helped in the inhibiting of tumor growth compared to an equivalent dose of free doxorubicin which proved ineffective otherwise (Tian et al., 2014). Exosomes released by macrophages have been loaded with paclitaxel (a drug used to treat multi drug resistant cancer) and used to treat mouse 3LL-M27 lung carcinoma cells along with drug resistant and wild type strains of MDCK cells *in vitro* and C57Bl/6 mice bearing pulmonary metastases *in vivo* showing increased cytotoxicity against the lung cancer cell lines *in vitro* and reduced tumor size *in vivo*.

In addition to drug, MVs have also been tapped for therapeutic delivery of proteins. MV contain a significant number of binding sites for specific targeting/homing ligands for targeted protein (Yang et al., 2019). As such, therapeutic proteins can be delivered by engineering the MV surface using the fusion of the moiety of interest adding to the ever increasing repertoire of smart engineered membrane vesicles for precision medicine (Tran et al., 2020) and have been extensively reviewed by Xing et al*.* (2020). Similarly, MVs harbor excellent potential for targeted delivery of nucleic acids while obviating issue of degradation and have been reported extensively over the years (Alvarez-Erviti et al., 2011). Injection of Exosomes derived from marrow stromal cell (MSC) after the transfection with miR-146b miRNA, into the primary brain tumor of rats showed significant reduction in the glioma xenograft growth suggesting a new treatment strategy for malignant glioma (Katakowski et al., 2013). Similarly, stellate cell derived EVs loaded with miRNA-335-5p when delivered to hepatocellular carcinoma cells inhibited cancer growth and invasion both *in vitro* and *in vivo*. It also led to the shrinking of the HCC and also the identification of mRNA down regulated due to the miRNA such as CDC42, CDK2, EIF2C2, CSNK1G2, ZMYND8 and others that have been implicated in hepatocellular carcinoma (Wang et al., 2018). In recent years, Levenberg et al. using mesenchymal stem cell derived exosomes loaded with phosphatase and tensin homolog small interfering RNA (ExoPTEN) showed robust axonal outgrowth of neurons *in vitro*, and improved neovascularization and therapeutic efficacy in rats with spinal cord injury (Guo et al., 2019). In an excellent study, reprogramming of native MVs for RNA delivery was elucidated. An arrow-shaped RNA to control ligand display on MV membranes for specific cell targeting was installed. Placement of membrane anchoring cholesterol at the tail of the arrow resulted in the display of RNA aptamer or folate on the outer surface of MVs; the resulting MVs were able to specifically deliver siRNA to cells, and halt tumor growth in prostate, breast and colorectal cancer models (Pi et al., 2018).

Stem cells have been shown to hold great promise for treating several diseases, and hence stem cell derived MVs are emerging as attractive modality for tissue generation. For example, perivascular stem cells (PSCs) derived MVs facilitate issue repair of perivascular stem cells while providing an ‘off-the-shelf' substitute for bone tissue regeneration (Xu et al., 2019). Interestingly MVs derived from human dermal fibroblasts have been shown to exhibit anti-skin-aging abilities (Hu et al., 2019). Similarly, lung spheroid cell secreted exosomes have been shown to be effective in different models of lung injury and fibrosis. Attenuation of lung fibrosis by re-establishing normal alveolar structure and decreasing collagen accumulation and myofibroblast proliferation indicate their great innate therapeutic potential (Dinh et al., 2020)**.**

MVs are advantageous due to their low immunogenicity and innate ability to interact with target cells owing to their biocompatibility which is an important pre-requisite for clinical applications. Other factors that propel MVs based clinical use are high stability in biological fluids, cell-cell communication, active targeting by modification/functionalization, suitability for multidrug delivery and a wide range ofdrug encapsulation methods. However, MV-based therapeutics have their own challenges (Ayala-Mar et al., 2019) such as sourcing of an optimal donor cell type, preserving the structural integrity of the MV, large-scale production, storage and high purity (Akuma et al., 2019) are some roadblocks for attaining a more practical means of exploiting MVs for theranostic use. In addition, poorly defined compositions and uptake mechanisms, lack of good manufacturing practice standards, in ability to translocate through renal barriers and in-depth lack of pre-clinical evaluation are some impediments that hamper heightened clinical transition of MVs based therapy. Further, engineered MVs require the precise evaluation of the interaction of the MVs with cells in order to determine their uptake routes. Finally, the route of exosome delivery is another key factor that affects the therapeutic application of exosomes as intravenous admiration has shown to lead to the faster clearance of the exosomes and lead to their accumulation in either the liver, spleen or kidney (Grange et al., 2014; Wiklander et al., 2015). This leads to the limited systemic admiration. To circumvent this issue reports have shown the alteration in the ligand of exosomes lead to enhanced target accumulation with certain limitations pertaining to the overall quantity of exosomes in the target organ (Ohno et al., 2013). Nonetheless MVs present new paradigms for diagnosis and prognosis against various diseases.

## Are There Other Innovative Uses of MVs BEYOND THERAPEUTICS AND DIAGNOSIS?

### A Complete Scaffold for Investigating Native Plasma Membrane Organization

#### Why Is Membrane Organization Important?

Plasma membrane is a key component of eukaryotic cells that orchestrates various membrane-associated cellular processes by selective recruitment and sorting of lipids and proteins into distinct spatially organized lipid domains. The most investigated lipid domains central to signal transduction and membrane trafficking pathways are the lipid rafts. Lipid rafts are conceived as dynamic, transient and heterogeneous membrane platforms enriched in cholesterol, glycosphingolipids such as SM and other saturated lipids in addition to raftophillic proteins. The functional implication of lipid rafts and other specific lipid domains has been demonstrated in regulating lipid-lipid and lipid protein interactions critical for signaling, trafficking, cell growth, migration, pathogen entry, and immune response. Moreover, structural and compositional fluctuations in plasma membrane domains govern membrane biophysical properties, such as order, fluidity, adhesion, elasticity, and lateral organization, control lipid/protein diffusion (Kreutzberger et al., 2019) lipid/protein interactions (Wang et al., 2016) and activities (Lorent et al., 2017). In fact, these biophysical properties dictate signaling efficiency and have recently been implicated in cancer, inflammatory conditions, and infectious diseases. Hence, lipid domains have held center stage in membrane biology and biophysics. However, experimentally intractable inherent complexities of the native biological membranes impede obtaining much-sought-after insights into membrane structure and function. For instance, the three-dimensional morphology below optical resolution and highly dynamic lipid domain fluctuations restrict the use of fluorescence microscopy and related advanced spectroscopic and microscopic methods.

#### What the Various Available Tools for Exploring Membrane Structure?

One obvious solution to query native membrane structure and function is the reconstitution of membrane-related processes outside the cell using artificial model membrane systems of manually tunable compositions. Well–established systems include detergent resistant membrane fractions (DRMs) (Schroeder et al., 1994), solid supported bilayers and vesicles of varying sizes; large, small, and giant vesicles (Chan and Boxer, 2007). Due to their robust nature, small and large vesicles can be handled like any aqueous solutions and hence are used in experiments that depend on ensemble measurements for readout like fluorescence and infrared spectroscopy. Giant vesicles, also known as giant unilamellar vesicles (GUVs) with their larger size above the diffraction limit permit their use for microscopy-based measurements. Other salient features of GUVs include low intrinsic curvature, tunable membrane tension by regulation of osmotic tension, and utility for measuring lateral lipid diffusion due to their quasi-planar geometry compatible with the spatial scale of confocal microscopy. However certain caveats such as limited lipid compositional complexity lack of cellular structures, altered lipid asymmetry in bilayer leaflets, and challenges in reconstituting membrane proteins, impede tapping the full potential of the above model membrane systems in a quest to understand the tightly regulated membrane organization and hence functions.

Interestingly, many of these limitations have been addressed by the development of cell-derived giant plasma membrane vesicles (GPMVs) that readily recapitulates the native integrity of biological membranes. GPMVs are produced by chemically induced blebbing of cell plasma membranes and offer genetic controllability of fine-tuning their lipid and protein compositions. One of the earliest mentioned method for membrane blebbing was reported for sarcoma cells using sulfhydryl group based chemicals (Belkin and Hardy, 1961). More recent accounts are the production of either cell attached GPMVs or cell free GPMVs using a variety of chemicals (Scott and Maercklein, 1979; Baumgart et al., 2007). The generic mechanism of GPMVs formation involves local decoupling of plasma membrane from the underlying cytoskeleton by dissociation of protein-lipid/protein interactions, followed by contraction of the actomyosin cortex that propels the cytoplasmic fluid to fill the expanding membrane area and eventually lead to retraction of the bleb.

#### How Are MVs Bridging the Gap in Our Understanding of Complex Cellular Membranes?

**FIGURE 4:**
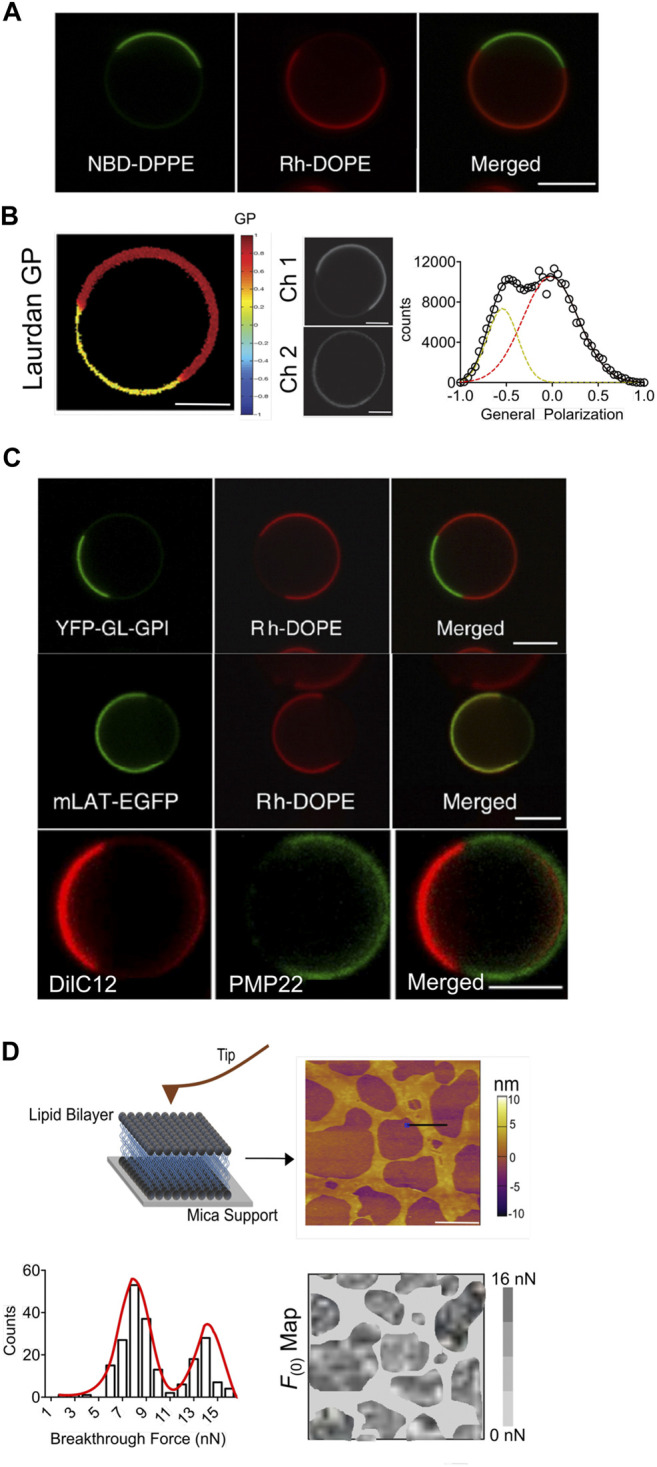
Giant plasma membrane vesicles for investigating membrane organization and proteins localization. **(A)** Fluorescent images showing phase segregation into liquid ordered (l_o_) and liquid disordered (l_d_) regions in cell-derived GPMVs labeled with NDB-DPPE (green, preferentially partitions into l_o_) and Rh-DOPE (red, partitions into l_d_), scale bar: 5 μm. Image reprinted from Biochimica et Biophysica Acta (BBA), Biomembranes, Sengupta, P *et al.* Structural determinants for partitioning of lipids and proteins between coexisting fluid phases in giant plasma membrane vesicles, 2008, 1778, 20–32. Copyright (2008), with permission from Elsevier **(B)** Representative Laurdan two photon microscopy images of GPMVs derived from A431 cells. Ch1 denotes the emission from Laurdan at 425/50 nm and ch2 at 525/70 nm, with GP image calculated using (Ch1- G * Ch2)/(Ch1+ G * Ch2) in the region. G denotes the calibration factor, scale bar 10 μm. GP scale ranges from −1.0 to +1.0. Image reproduced with permission from Kaiser, HJ et al*.* Proceedings of the National Academy of Sciences, 2009, 106 (39) 16,645–16,650. The GP values at each pixel are extracted and corresponding GP histogram is generated that shows bimodal GP distribution indicative of at least two lipid phases with distinct order. **(C)** Representative fluorescence images showing partitioning of GPI-anchored protein (green, YFP-GL-GPI) and transmembrane protein (green, mLAT-EGFP) in the l_o_ and l_d_ region, respectively, within phase segregated GMPVs. The GMPVs were also labeled with l_d_ marker (red, Rh-DOPE). Image reprinted from Biochimica et Biophysica Acta (BBA) - Biomembranes, Sengupta, P. et al*.* Structural determinants for partitioning of lipids and proteins between coexisting fluid phases in giant plasma membrane vesicles, 2008, 1778, 20–32. Copyright (2008), with permission from Elsevier Representative fluorescence images showing partitioning of PMP22 (green) into the l_o_ region of phase segregated GPMVs derived from Hela cells. The GPMVs were also labeled with l_d_ marker (red, DilC12). Image reproduced with permission from Marinko, JT et al*.* Proceedings of the National Academy of Sciences, 2020, 117 (25) 14,168–14,177. Copyright (2020) Marinko, Kenworthy, Sanders **(D)** Schematic representation of atomic force microscopy and spectroscopy workflow for studying mechanical membrane properties. Left, solid supported bilayer (SSB) formed from GPMVs fusion on solid mica support. Topographical AFM image of such a SSB and the corresponding force maps, depicting breakthrough force distribution across bilayer surface. AFM Image reprinted from Biopyhiscal Journal, Adhyapak, P. et al*.* Dynamical Organization of Compositionally Distinct Inner and Outer Membrane Lipids of Mycobacteria, 2020, 118, 1,279–1,291. Copyright (2020), with permission from Elsevier. Breakthrough force corresponds to force required to pierce through the bilayer surface. Histogram depiction of the breakthrough force distribution showing bimodal force distribution indicative of at least two mechanically distinct lipid regions.

Baumgart et al. rendered the seminal study that embarked the journey of GPMVs for studying lateral membrane organization by demonstrating lipid-lipid phase separation in GPMVs, reminiscent of lipid ordered and lipid-disordered domains (Figure 4A). Particularly was the validation of the most important lipid raft tenets: selective sorting of specific lipids and proteins such as GPI-anchored proteins and the glycosphingolipid GM1 into one of the GPMVs liquid phases and dependence of lipid phase separation on membrane cholesterol (Steinkühler et al., 2019). Addition of distinct diffusivity (Levental et al., 2009) and membrane order (Kaiser et al., 2009) profiles for the coexisting domains in GPMVs confirmed the liquid- ordered/liquid-disordered phase immiscibility to exist in natural cell membranes and subsequently in GPMVs originating from these (Sezgin et al., 2015), (Figure 4B). Further, using a panel of single-pass transmembrane domain proteins with varying lengths, a direct correlation of their partitioning into lipid rafts in GPMVs reinforced the notion of lateral heterogeneities in plasma membranes (Diaz-Rohrer et al., 2014). More recently, label free cryo-EM has shown similar nanoscale lateral heterogeneities namely, membrane thickness and molecular density, in isolated GPMVs (Heberle et al., 2020). This study also highlights the application of probe-free imaging of nanodomains in unperturbed membranes. The most celebrated use of GPMVs has been its use for studying protein partitioning to decipher lipid-mediated cues to regulate protein localization and function and is extensively reviewed in (Levental and Levental, 2015). The work from the group of Barbara Baird has demonstrated the use of GPMVs for investigating selective partitioning of proteins to decipher the structural basis of their membrane partitioning. For instance, using fluorescent GPI anchored proteins, YFP-GL-GPI (dimeric) and mYFP-GL-GPI (monomeric), they revealed the selective localization of GPI-anchored proteins into the liquid ordered GPMVs phases dictated by the saturated nature of the GPI membrane anchor (Sengupta et al., 2008) (Figure 4C). Furthermore, they were able to show that YFP dimerization does not perturb membrane partitioning of YFP-GL-GPI proteins. In contrast to GPI-anchored protein (also peripheral proteins), the authors showed that aggregation of transmembrane proteins, such as LAT, does influence their membrane localization. That is, while monomeric LAT (mLAT) prefers liquid disordered phase (Figure 4C), the dimeric LAT partition similarly into both the phases in phase-segregated GPMVs. The authors thus demonstrated that the membrane anchor of peripheral and transmembrane proteins and their aggregation states dictates their localization preference to ordered or disordered lipid domains within GPMVs (Figure 4C). A recent example for the usage of phase segregated GPMVs for studying partitioning of transmembrane proteins was reported by Marinko et al. (2020). They demonstrated the helical tetraspan peripheral myelin protein 22 (PMP22) localizes into the ordered domains of GPMVs formed from HeLa cells and primary Schwann cell. Introducing a L16P mutation in the TM1 helical segment reversed the phase preference (Figure 4C). It was also noticed that GPMVs containing the WT PMP22 exhibited a higher T_misc_ than the ones with the L16P mutation implying the stabilization effect of the protein on the ordered membrane domains (Marinko et al., 2020). These are few such examples from an array of discoveries that have used GPMVs to understand lipid phase mediated protein localization, partitioning and effects of such specificities (Sengupta et al., 2019; Capone et al., 2020; Castello-Serrano et al., 2020).

One seminal example of the use of GPMVs is for understanding the mechanism of entry and the phase preference for the binding and fusion of HIV virions with the host cell. As fusion is an import process in the initiation of HIV entry, it was discovered that phase boundaries between l_o_/l_d_ domains provided the driving force for fusion (Yang et al., 2016). In this study, membrane vesicles from HeLa cells containing natural receptors were used to show that the HIV gp120 binds the CD4 receptors located in dynamic cholesterol rich lipid domains. If this CD4 receptor was located near a CCR5 co-receptor at these phase boundaries, the initial binding led to structural changes of gp120. Post binding to CD4 and CCR5 the fusion peptide is exposed owing to the conformational changes in gp41 (Yang et al., 2017c). This led to further targeting of the fusion peptide to the same fusion promoting areas on the host membrane. This opened up new ways in which GPMVs could be used to screen viral entry inhibitors in the future.

Moreover, nano mechanical properties of membrane regulate cellular adhesions and migration and GPMVs based solid supported bilayers in combination with force spectroscopy provides ideal platform to investigate these aspects in near native lipid and protein environment (Tian et al., 2014) (Figure 4D). GPMVs have also being exploited as novel immunoresponsive drug delivery platforms. For instance, GPMVs derived from tumour tissue of BALB/c or C57Bl/6 mice, studded with a breast cancer antigen HER-2 (Patel et al., 2016), GPI-HER-2-PMVs, elicited a strong immune response along with offering protection against tumor challenge. Further, a Th1-type and Th2-type antibody responses was also generated elucidating engineered GPMVs as efficient single antigen delivery system against numerous types of cancer.

Taking inspiration from PM derived GPMVs, very recently giant vesicles derived from endoplasmic reticulum (GERS), have been developed. This proof-of-concept study by Grimmer et al. revealed GERS as an attractive and novel tool to elucidate ER-regulated process. By demonstrating the compatibility of various biophysical techniques on the newly developed membrane mode along with ability to incorporate additional fluorescent fused proteins of interest into the ER by genetic means post GERs production, the authors revealed the potential application of these systems to investigate enzyme functions and lipid metabolism regulated by ER membrane; a field less explored. Notably, in parallel, another studies revealed the ability of ER membranes to exhibit temperature-dependent phase segregation, similar to that seen in plasma membrane (King et al., 2020) as well as the ability of these phases to selectively sort specific ER proteins (Bag et al., 2020). This has a huge implication on various ER-regulated cellular processes and the use of GERVs to address these concerns remains to be investigated.

Finally, the study of lipid rafts have been extensively investigated in eukaryotic cells, however evidence indicating their presence in bacterial is reinforcing the rather ubiquitous nature of lipid domains in regulating cellular processes across various living forms (Allen et al., 2016; García-Fernández et al., 2017; Raghunathan and Kenworthy, 2018; Toledo et al., 2018; Adhyapak et al., 2020). In this regard, bacterial OMVs serve as valuable tools for addressing the functional role of membrane liquid-liquid phase separation in dictating bacterial physiology, response to therapeutic agents, and in interactions with the host during infection (Toyofuku et al., 2019). Of note, bacterial OMVs for studying the structure and function of outer membrane proteins (OMPs)-the most sought-after therapeutic targets in bacterial infections is also gaining speed (Thoma et al., 2018; Thoma and Burmann, 2020).

GPMVs, thus have proven a versatile system for studying plasma membrane organization dictated processes; lipid phase segregation, distribution of lipids and membrane proteins into distinct domains, protein sorting, regulation of protein function, and membrane fusion. However, there are certain limitations that signal caution in analyzing data collected with GPMVs. These are the lack of perfect plasma membrane asymmetry (Keller et al., 2009), effect of chemicals used during GPMV production (Steinkühler et al., 2019) and lack of cytoskeletal features; the latter has been partially addressed by polymerizing actin within GPMVs to construct actin filament network underneath the lipid bilayer. Nonetheless, even with these caveats, GPMVs are still the most immediately relevant model membrane systems to investigate native membranes, until advances in biophysics, lipid chemical biology and biochemistry enable querying relevant membrane centric question in native live cells.

## Conclusion

Secretion of membrane vesicles is now a well-accepted phenomenon across various life forms and plays crucial roles in normal cell functioning. They serve varied functional roles, such as in communication of signals between and within a cell by decorating their surface with specific lipid and protein components. Consequently, they have occupied a center stage in disease biology focused on 1) discovery of pathogenic mechanisms at the interface of host and pathogen, 2) to facilitate effective and non-invasive source of biomarkers (Kim et al., 2020), and 3) serve as biocompatible scaffold for therapeutic delivery and vaccine candidates (Tanziela et al., 2020). Apart from these, another judicious application of membrane vesicle has been in the studies on lateral membrane organization and its functional relevance. The ability to combine the complexities of the near native cell membranes with the applicability towards various microscopic and spectroscopic techniques have provided unprecedented insights into the orchestrating functions of lipids within membrane domains in channelizing protein and lipid recruitment and subsequently fine-tuning their function implicit in cellular signaling (Schneider et al., 2017; Worch et al., 2017; Marinko et al., 2020). In future, this field would benefit by in-depth quantitative lipidomic and proteomic analysis to delineate their regulatory controls in cellular signaling, communication and decipher critical nodes and hence diagnostic avenues in pathogenesis of various diseases.

## Glossary

AHLs: L-homoserine lactones
APCs: Antigen Presenting Cells
CE: Cholesteryl ester
Cer: Ceramide
Chol: Cholesterol
CL: Cardiolipin
CRC: Colorectal cancer
DAG: Diacylglycerol
D-OMV: Detergent extracted Outer Membrane Vesicles
DRMs: Detergent Resistant Membrane fractions
ER: Endoplasmic Reticulum
ESCRT: Endosomal Sorting Complexes Required for Transport
GERVs: Giant Endoplasmic Reticulum Vesicles
GPMVs: Giant Plasma Membrane Vesicles
GS: Gangliosides
GUVs: Giant Unilamellar Vesicles
HexCer: Hexosylceramide
HSL: Homoserine lactone
LacCer: Lactosylceramide
LAM: Lipoarabinomannan
LPC: lysophosphatidylcholine
LPS: lipopolysaccharide
Lyso-PC: lyso-phosphatidylcholine
Lyso-PE: lyso-phosphatidylethanolamine
Lyso-PI: lyso-Phosphatidylinositol
Lyso-PS: lyso-phosphatidylserine
MAG: Monoacylglycerol
Mtb: Mycobacterium tuberculosis
MVBs: Multivesicular Bodies
MVs: Membrane Vesicles
Nm: Neisseria meningitides
NOD: Nucleotide binding Oligomerization Domain
N-WASP: Neural Wiskott-Aldrich Syndrome Protein
O-IMV: Outer-Inner Membrane Vesicles
OMPs: Outer Membrane Proteins
OMV: Outer Membrane Vesicles
PC: Phosphatidylcholine
PE: Phosphatidylethanolamine
PG: Phosphatidylglycerol
PI: Phosphatidylinositol
PNAG: β(1-6)-N-acetylglucosamine
PQS: Pseudomonas Quinolone Signal
PS: Phosphatidylserine
QS: Quorum Sensing
ROS: Reactive Oxygen Species
SM: Sphingomyelin
TAG: Triacylglycerol
TDM: Trehalose dimycolate
TG: Triglyceride
TLR: Toll-like Receptor.

## References

[B1] Abramowicz A. Wojakowska A. Marczak L. Lysek-Gladysinska M. Smolarz M. Story M. D. (2019). Ionizing radiation affects the composition of the proteome of extracellular vesicles released by head-and-neck cancer cells *in vitro* . J. Radiat. Res. 60 (3), 289–297. 10.1093/jrr/rrz001 30805606 PMC6530623

[B2] Adhyapak P. Srivatsav A. T. Mishra M. Singh A. Narayan R. Kapoor S. (2020). Dynamical organization of compositionally distinct inner and outer membrane lipids of mycobacteria. Biophys. J. 118, 1279–1291. 10.1016/j.bpj.2020.01.027 32061274 PMC7091463

[B3] Akuma P. Okagu O. D. Udenigwe C. C. (2019). Naturally occurring exosome vesicles as potential delivery vehicle for bioactive compounds. Front. Sustain. Food Syst. 3. 10.3389/fsufs.2019.00023

[B4] Alaniz R. C. Deatherage B. L. Lara J. C. Cookson B. T. (2007). Membrane vesicles are immunogenic facsimiles of *Salmonella typhimurium* that potently activate dendritic cells, prime B and T cell responses, and stimulate protective immunity in vivo. J. Immunol. 179, 7692–7701. 10.4049/jimmunol.179.11.7692 18025215

[B5] Allen R. C. McNally L. Popat R. Brown S. P. (2016). Quorum sensing protects bacterial co-operation from exploitation by cheats. ISME J. 10, 1706–1716. 10.1038/ismej.2015.232 26744811 PMC4918439

[B6] Allenson K. Castillo J. San Lucas F. A. Scelo G. Kim D. U. Bernard V. (2017). High prevalence of mutant KRAS in circulating exosome-derived DNA from early-stage pancreatic cancer patients. Ann. Oncol. 28, 741–747. 10.1093/annonc/mdx004 28104621 PMC5834026

[B7] Altman B. J. Stine Z. E. Dang C. V. (2016). From Krebs to clinic: glutamine metabolism to cancer therapy. Nat. Rev. Cancer 16, 619–634. 10.1038/nrc.2016.71 27492215 PMC5484415

[B8] Alvarez-Erviti L. Seow Y. Yin H. Betts C. Lakhal S. Wood M. J. (2011). Delivery of siRNA to the mouse brain by systemic injection of targeted exosomes. Nat. Biotechnol. 29, 341–345. 10.1038/nbt.1807 21423189

[B9] Arab S. Hadjati J. (2019). Adenosine blockage in tumor microenvironment and improvement of cancer immunotherapy. Immune Netw. 19 (4), e23. 10.4110/in.2019.19.e23 31501711 PMC6722273

[B10] Arantes T. S. Rodrigues R. A. dos Santos Silva L. K. Oliveira G. P. de Souza H. L. Khalil J. Y. (2016). The large marseillevirus explores different entry pathways by forming giant infectious vesicles. J. Virol. 90, 5246–5255. 10.1128/jvi.00177-16 26984730 PMC4934737

[B11] Athman J. J. Sande O. J. Groft S. G. Reba S. M. Nagy N. Wearsch P. A. (2017). Membrane vesicles inhibit T cell activation. J. Immunol. 198, 2028–2037. 10.4049/jimmunol.1601199 28122965 PMC5322216

[B12] Athman J. J. Wang Y. McDonald D. J. Boom W. H. Harding C. V. Wearsch P. A. (2015). Bacterial membrane vesicles mediate the release of *Mycobacterium tuberculosis* lipoglycans and lipoproteins from infected macrophages. J. Immunol. 195, 1044–1053. 10.4049/jimmunol.1402894 26109643 PMC4506856

[B13] Augustyniak D. Seredyński R. McClean S. Roszkowiak J. Roszniowski B. Smith D. L. (2018). Virulence factors of Moraxella catarrhalis outer membrane vesicles are major targets for cross-reactive antibodies and have adapted during evolution. Sci. Rep. 8. 10.1038/s41598-018-23029-7 PMC5862889 29563531

[B221] Ayala-Mar S. Donoso-Quezada J. Gallo-Villanueva R. C. Perez-Gonzalez V. H. González-Valdez J. (2019). Recent advances and challenges in the recovery and purification of cellular exosomes. Electrophoresis 40, 3036–3049. 10.1002/elps.201800526 31373715 PMC6972601

[B14] Bag N. Ramezani M. Holowka D. A. Baird B. A. (2020). Bringing light to ER contacts and a new phase in organelle communication. Proc. Natl. Acad. Sci. U. S. A. 117, 9668–9670. 10.1073/pnas.2003620117 32345722 PMC7211920

[B15] Bahar O. Mordukhovich G. Luu D. D. Schwessinger B. Daudi A. Jehle A. K. (2016). Bacterial outer membrane vesicles induce plant immune responses. Mol. Plant Microbe Interact 29, 374–384. 10.1094/MPMI-12-15-0270-R 26926999

[B16] Baumgart T. Hammond A. T. Sengupta P. Hess S. T. Holowka D. A. Baird B. A. (2007). Large-scale fluid/fluid phase separation of proteins and lipids in giant plasma membrane vesicles. Proc. Natl. Acad. Sci. USA 104, 3165–3170. 10.1073/pnas.0611357104 17360623 PMC1805587

[B17] Belkin M. Hardy W. G. (1961). Relation between water permeability and integrity of sulfhydryl groups in malignant and normal cells. J. Biophys. Biochem. Cytol. 9, 733–745. 10.1083/jcb.9.4.733 19866586 PMC2225047

[B18] Bhatnagar S. Shinagawa K. Castellino F. J. Schorey J. S. (2007). Exosomes released from macrophages infected with intracellular pathogens stimulate a proinflammatory response *in vitro* and *in vivo* . Blood 110, 3234–3244. 10.1182/blood-2007-03-079152 17666571 PMC2200902

[B19] Biller S. J. Schubotz F. Roggensack S. E. Thompson A. W. Summons R. E. Chisholm S. W. (2014). Bacterial vesicles in marine ecosystems. Science 343, 183–186. 10.1126/science.1243457 24408433

[B20] Bomberger J. M. MacEachran D. P. Coutermarsh B. A. Ye S. O'Toole G. A. Stanton B. A. (2009). Long-distance delivery of bacterial virulence factors by pseudomonas aeruginosa outer membrane vesicles. Plos Pathog. 5, e1000382. 10.1371/journal.ppat.1000382 19360133 PMC2661024

[B21] Brameyer S. Plener L. Müller A. Klingl A. Wanner G. Jung K. (2018). Outer membrane vesicles facilitate trafficking of the hydrophobic signaling molecule CAI-1 between Vibrio harveyi cells. J. Bacteriol. 200. 10.1128/JB.00740-17 PMC6040191 29555694

[B22] Brauer V. S. Pessoni A. M. Bitencourt T. A. de Paula R. G. de Oliveira Rocha L. Goldman G. H. (2020). Extracellular vesicles from Aspergillus flavus induce M1 polarization in vitro. mSphere 5. 10.1128/msphere.00190-20 PMC7203453 32376699

[B23] Bryant K. L. Mancias J. D. Kimmelman A. C. Der C. J. (2014). KRAS: feeding pancreatic cancer proliferation. Trends Biochem. Sci. 39, 91–100. 10.1016/j.tibs.2013.12.004 24388967 PMC3955735

[B24] Brzozowski J. S. Jankowski H. Bond D. R. McCague S. B. Munro B. R. Predebon M. J. (2018). Lipidomic profiling of extracellular vesicles derived from prostate and prostate cancer cell lines. Lipids Health Dis. 17, 211. 10.1186/s12944-018-0854-x 30193584 PMC6128989

[B25] Budhani R. K. Struthers J. K. (1998). Interaction of Streptococcus pneumoniae and Moraxella catarrhalis: investigation of the indirect pathogenic role of beta-lactamase-producing moraxellae by use of a continuous-culture biofilm system. Antimicrob. Agents Chemother. 42, 2521–2526. 10.1128/aac.42.10.2521 9756750 PMC105877

[B26] Capone R. F. Tiwari A. Fricke N. Hadziselimovic A. Kenworthy A. K. Sanders C. R. (2020). Use of giant plasma membrane vesicles (GPMV) to examine the lo/ld phase preference of the C99 domain of the amyloid precursor protein. Biophys. J. 118, 392a. 10.1016/j.bpj.2019.11.2231

[B27] Castello-Serrano I. Lorent J. H. Ippolito R. Levental K. R. Levental I. (2020). Myelin-associated MAL and PLP are unusual among multipass transmembrane proteins in preferring ordered membrane domains. J. Phys. Chem. B 124, 5930–5939. 10.1021/acs.jpcb.0c03028 32436385 PMC7792449

[B28] Cazzoli R. Buttitta F. Di Nicola M. Malatesta S. Marchetti A. Rom W. N. (2013). MicroRNAs derived from circulating exosomes as noninvasive biomarkers for screening and diagnosing lung cancer. J. Thorac. Oncol. 8, 1156–1162. 10.1097/JTO.0b013e318299ac32 23945385 PMC4123222

[B29] Cestari I. Ansa-Addo E. Deolindo P. Inal J. M. Ramirez M. I. (2012). Trypanosoma cruzi immune evasion mediated by host cell-derived microvesicles. J. Immunol. 188, 1942–1952. 10.4049/jimmunol.1102053 22262654

[B30] Chan Y. H. Boxer S. G. (2007). Model membrane systems and their applications. Curr. Opin. Chem. Biol. 11, 581–587. 10.1016/j.cbpa.2007.09.020 17976391 PMC2196400

[B31] Chattopadhyay M. K. Jaganandham M. V. (2015). Vesicles-mediated resistance to antibiotics in bacteria. Front. Microbiol. 6, 758. 10.3389/fmicb.2015.00758 26257725 PMC4511839

[B32] Chen I. H. Xue L. Hsu C. C. Paez J. S. Pan L. Andaluz H. (2017). Phosphoproteins in extracellular vesicles as candidate markers for breast cancer. Proc. Natl. Acad. Sci. USA 114, 3175–3180. 10.1073/pnas.1618088114 28270605 PMC5373352

[B33] Chen S. Datta-Chaudhuri A. Deme P. Dickens A. Dastgheyb R. Bhargava P. (2019). Lipidomic characterization of extracellular vesicles in human serum. J. Circ. Biomarkers 8. 10.1177/1849454419879848 PMC6769212 31632506

[B34] Cheng Y. Schorey J. S. (2013). Exosomes carrying mycobacterial antigens can protect mice against *Mycobacterium tuberculosis* infection. Eur. J. Immunol. 43, 3279–3290. 10.1002/eji.201343727 23943377 PMC4076847

[B35] Chowdhury C. Jagannadham M. V. (2013). Virulence factors are released in association with outer membrane vesicles of *Pseudomonas syringae* pv. tomato T1 during normal growth. Biochim. Biophys. Acta 1834, 231–239. 10.1016/j.bbapap.2012.09.015 23043909

[B36] Ciardiello C. Leone A. Lanuti P. Roca M. S. Moccia T. Minciacchi V. R. (2019). Large oncosomes overexpressing integrin alpha-V promote prostate cancer adhesion and invasion via AKT activation. J. Exp. Clin. Cancer Res. 38, 317. 10.1186/s13046-019-1317-6 31319863 PMC6639931

[B37] Clos-Garcia M. Loizaga-Iriarte A. Zuñiga-Garcia P. Sánchez-Mosquera P. Rosa Cortazar A. González E. (2018). Metabolic alterations in urine extracellular vesicles are associated to prostate cancer pathogenesis and progression. J. Extracell. Vesicles 7, 1470442. 10.1080/20013078.2018.1470442 29760869 PMC5944373

[B38] Codemo M. Muschiol S. Iovino F. Nannapaneni P. Plant L. Wai S. N. (2018). Immunomodulatory effects of pneumococcal extracellular vesicles on cellular and humoral host defenses. MBio 9. 10.1128/mBio.00559-18 PMC5893880 29636428

[B39] Colombo M. Giannandrea D. Lesma E. Basile A. Chiaramonte R. (2019). Extracellular vesicles enhance multiple myeloma metastatic dissemination. Int. J. Mol. Sci. 20 (13), 3236. 10.3390/ijms20133236 PMC6650870 31266187

[B40] Dai J. Escara-Wilke J. Keller J. M. Jung Y. Taichman R. S. Pienta K. J. (2019). Primary prostate cancer educates bone stroma through exosomal pyruvate kinase M2 to promote bone metastasis. J. Exp. Med. 216 (12), 2883–2899. 10.1084/jem.20190158 31548301 PMC6888980

[B41] De Palma M. Biziato D. Petrova T. V. (2017). Microenvironmental regulation of tumour angiogenesis. Nat. Rev. Cancer 17, 457–474. 10.1038/nrc.2017.51 28706266

[B42] De Toro J. Herschlik L. Waldner C. Mongini C. (2015). Emerging roles of exosomes in normal and pathological conditions: new insights for diagnosis and therapeutic applications. Front. Immunol. 6, 203. 10.3389/fimmu.2015.00203 25999947 PMC4418172

[B43] Deatherage B. L. Cookson B. T. (2012). Membrane vesicle release in bacteria, eukaryotes, and archaea: a conserved yet underappreciated aspect of microbial life. Infect. Immun. 80, 1948–1957. 10.1128/IAI.06014-11 22409932 PMC3370574

[B44] DeBerardinis R. J. Chandel N. S. (2016). Fundamentals of cancer metabolism. Sci. Adv. 2, e1600200. 10.1126/sciadv.1600200 27386546 PMC4928883

[B45] Diaz-Rohrer B. B. Levental K. R. Simons K. Levental I. (2014). Membrane raft association is a determinant of plasma membrane localization. Proc. Natl. Acad. Sci. USA 111, 8500–8505. 10.1073/pnas.1404582111 24912166 PMC4060687

[B46] Dinh P. U. C. Paudel D. Brochu H. Popowski K. D. Gracieux M. C. Cores J. (2020). Inhalation of lung spheroid cell secretome and exosomes promotes lung repair in pulmonary fibrosis. Nat. Commun. 11. 10.1038/s41467-020-14344-7 PMC7048814 32111836

[B47] Faict S. Oudaert I. D’auria L. Dehairs J. Maes K. Vlummens P. (2019). The transfer of sphingomyelinase contributes to drug resistance in multiple myeloma. Cancers (Basel) 11 (12), 1823. 10.3390/cancers11121823 PMC6966559 31756922

[B48] Fais S. O'Driscoll L. Borras F. E. Buzas E. Camussi G. Cappello F. (2016). Evidence-based clinical use of nanoscale extracellular vesicles in nanomedicine. ACS Nano 10, 3886–3899. 10.1021/acsnano.5b08015 26978483

[B49] Fan Z. Xiao K. Lin J. Liao Y. Huang X. (2019). Functionalized DNA enables programming exosomes/vesicles for tumor imaging and therapy. Small 15, e1903761. 10.1002/smll.201903761 31614072

[B50] Fong M. Y. Zhou W. Liu L. Alontaga A. Y. Chandra M. Ashby J. (2015). Breast-cancer-secreted miR-122 reprograms glucose metabolism in premetastatic niche to promote metastasis. Nat. Cel Biol. 17, 183. 10.1038/ncb3094 PMC4380143 25621950

[B51] Freitas M. S. Bonato V. L. D. Pessoni A. M. Rodrigues M. L. Casadevall A. Almeida F. (2019). Fungal extracellular vesicles as potential targets for immune interventions. mSphere 4. 10.1128/msphere.00747-19 PMC6835212 31694899

[B52] Gao X. Li S. Ding F. Fan H. Shi L. Zhu L. (2019). Rapid detection of exosomal MicroRNAs using virus-mimicking fusogenic vesicles. Angew. Chem. - Int. Ed. 58, 8719–8723. 10.1002/anie.201901997 31095853

[B53] García-Fernández E. Koch G. Wagner R. M. Fekete A. Stengel S. T. Schneider J. (2017). Membrane microdomain disassembly inhibits MRSA antibiotic resistance. Cell 171, 1354–1367. 10.1016/j.cell.2017.10.012 e20 29103614 PMC5720476

[B54] Giuliani M. M. Adu-Bobie J. Comanducci M. Aricò B. Savino S. Santini L. (2006). A universal vaccine for serogroup B meningococcus. Proc. Natl. Acad. Sci. USA 103, 10834–10839. 10.1073/pnas.0603940103 16825336 PMC2047628

[B55] Glover S. C. Nouri M. Tuna K. M. Mendoza Alvarez L. B. Ryan L. K. Shirley J. F. (2019). Lipidomic analysis of urinary exosomes from hereditary α-tryptasemia patients and healthy volunteers. FASEB Bioadv 1, 624–638. 10.1096/fba.2019-00030 31803861 PMC6892164

[B56] Grange C. Tapparo M. Bruno S. Chatterjee D. Quesenberry P. J. Tetta C. (2014). Biodistribution of mesenchymal stem cell-derived extracellular vesicles in a model of acute kidney injury monitored by optical imaging. Int. J. Mol. Med. 33, 1055–1063. 10.3892/ijmm.2014.1663 24573178 PMC4020482

[B57] Grimmer M. Bacia K. (2020). Giant Endoplasmic Reticulum vesicles (GERVs), a novel model membrane tool. Sci. Rep. 10. 10.1038/s41598-020-59700-1 PMC7033103 32080222

[B58] Guo S. Perets N. Betzer O. Ben-Shaul S. Sheinin A. Michaelevski I. (2019). Intranasal delivery of mesenchymal stem cell derived exosomes loaded with phosphatase and tensin homolog siRNA repairs complete spinal cord injury. ACS Nano 13, 10015–10028. 10.1021/acsnano.9b01892 31454225

[B59] Haraszti R. A. Didiot M. C. Sapp E. Leszyk J. Shaffer S. A. Rockwell H. E. (2016). High-resolution proteomic and lipidomic analysis of exosomes and microvesicles from different cell sources. J. Extracell. Vesicles 5, 32570. 10.3402/jev.v5.32570 27863537 PMC5116062

[B60] Hay N. (2016). Reprogramming glucose metabolism in cancer: can it be exploited for cancer therapy?. Nat. Rev. Cancer 16, 635–649. 10.1038/nrc.2016.77 27634447 PMC5516800

[B61] Heberle F. A. Doktorova M. Scott H. L. Skinkle A. D. Waxham M. N. Levental I. (2020). Direct label-free imaging of nanodomains in biomimetic and biological membranes by cryogenic electron microscopy. Proc. Natl. Acad. Sci. U. S. A. 117, 19943–19952. 10.1073/PNAS.2002200117 32759206 PMC7443941

[B62] Hough K. P. Wilson L. S. Trevor J. L. Strenkowski J. G. Maina N. Kim Y. I. (2018). Unique lipid signatures of extracellular vesicles from the airways of asthmatics. Sci. Rep. 8, 10340. 10.1038/s41598-018-28655-9 29985427 PMC6037776

[B63] Hu S. Li Z. Cores J. Huang K. Su T. Dinh P. U. (2019). Needle-free injection of exosomes derived from human dermal fibroblast spheroids ameliorates skin photoaging. ACS Nano 13, 11273–11282. 10.1021/acsnano.9b04384 31449388 PMC7032013

[B64] Huang W. Lin Y. Yi S. Liu P. Shen J. Shao Z. (2012). QsdH, a novel AHL lactonase in the RND-type inner membrane of marine pseudoalteromonas byunsanensis strain 1A01261. PLoS One 7, e46587. 10.1371/journal.pone.0046587 23056356 PMC3466314

[B65] Irene C. Fantappiè L. Caproni E. Zerbini F. Anesi A. Tomasi M. (2019). Bacterial outer membrane vesicles engineered with lipidated antigens as a platform for *Staphylococcus aureus* vaccine. Proc. Natl. Acad. Sci. U. S. A. 116, 21780–21788. 10.1073/pnas.1905112116 31591215 PMC6815149

[B66] Ismail S. Hampton M. B. Keenan J. I. (2003). *Helicobacter pylori* outer membrane vesicles modulate proliferation and interleukin-8 production by gastric epithelial cells. Infect. Immun. 71, 5670–5675. 10.1128/IAI.71.10.5670-5675.2003 14500487 PMC201067

[B67] Jeon J. Park S. C. Her J. Lee J. W. Han J. K. Kim Y. K. (2018). Comparative lipidomic profiling of the human commensal bacterium: propionibacterium acnes and its extracellular vesicles. RSC Adv. 8, 15241–15247. 10.1039/c7ra13769a PMC9080044 35541326

[B68] Josson S. Gururajan M. Sung S. Y. Hu P. Shao C. Zhau H. E. (2015). Stromal fibroblast-derived miR-409 promotes epithelial-to-mesenchymal transition and prostate tumorigenesis. Oncogene 34, 2690–2699. 10.1038/onc.2014.212 25065597

[B69] Kaiser H. J. Lingwood D. Levental I. Sampaio J. L. Kalvodova L. Rajendran L. (2009). Order of lipid phases in model and plasma membranes. Proc. Natl. Acad. Sci. USA 106, 16645–16650. 10.1073/pnas.0908987106 19805351 PMC2757813

[B70] Kalra H. Simpson R. J. Ji H. Aikawa E. Altevogt P. Askenase P. (2012). Vesiclepedia: a compendium for extracellular vesicles with continuous community annotation. Plos Biol. 10, e1001450. 10.1371/journal.pbio.1001450 23271954 PMC3525526

[B71] Katakowski M. Buller B. Zheng X. Lu Y. Rogers T. Osobamiro O. (2013). Exosomes from marrow stromal cells expressing miR-146b inhibit glioma growth. Cancer Lett. 335, 201–204. 10.1016/j.canlet.2013.02.019 23419525 PMC3665755

[B72] Keller H. Lorizate M. Schwille P. (2009). PI(4,5)P2 degradation promotes the formation of cytoskeleton-free model membrane systems. ChemPhysChem 10, 2805–2812. 10.1002/cphc.200900598 19784973

[B73] Kim D. K. Kang B. Kim O. Y. Choi D. S. Lee J. Kim S. R. (2013). EVpedia: an integrated database of high-throughput data for systemic analyses of extracellular vesicles. J. Extracell. Vesicles 2. 10.3402/jev.v2i0.20384 PMC3760654 24009897

[B74] Kim D. K. Lee J. Simpson R. J. Lötvall J. Gho Y. S. (2015). EVpedia: a community web resource for prokaryotic and eukaryotic extracellular vesicles research. Semin. Cel Dev. Biol. 40, 4–7. 10.1016/j.semcdb.2015.02.005 25704310

[B75] Kim O. Y. Park H. T. Dinh N. T. H. Choi S. J. Lee J. Kim J. H. (2017). Bacterial outer membrane vesicles suppress tumor by interferon-γ-mediated antitumor response. Nat. Commun. 8, 626. 10.1038/s41467-017-00729-8 28931823 PMC5606984

[B76] Kim S. Jeon O. H. Jeon Y. J. (2020). Extracellular RNA: emerging roles in cancer cell communication and biomarkers. Cancer Lett. 495, 33–40. 10.1016/j.canlet.2020.09.002 32916182

[B77] King C. Sengupta P. Seo A. Y. Lippincott-Schwartz J. (2020). ER membranes exhibit phase behavior at sites of organelle contact. Proc. Natl. Acad. Sci. U. S. A. 117, 7225–7235. 10.1073/pnas.1910854117 32179693 PMC7132286

[B78] Kreger B. T. Johansen E. R. Cerione R. A. Antonyak M. A. (2016). The enrichment of survivin in exosomes from breast cancer cells treated with paclitaxel promotes cell survival and chemoresistance. Cancers (Basel) 8 (12), 111. 10.3390/cancers8120111 PMC5187509 27941677

[B79] Kreutzberger A. J. B. Ji M. Aaron J. Mihaljević L. Urban S. (2019). Rhomboid distorts lipids to break the viscosity-imposed speed limit of membrane diffusion. Science 80, 363. 10.1126/science.aao0076 PMC6368390 30705155

[B80] Krishnamurthy A. McGrath J. Cripps A. W. Kyd J. M. (2009). The incidence of Streptococcus pneumoniae otitis media is affected by the polymicrobial environment particularly Moraxella catarrhalis in a mouse nasal colonisation model. Microbes Infect. 11, 545–553. 10.1016/j.micinf.2009.03.001 19306940

[B81] Kruh-Garcia N. A. Wolfe L. M. Chaisson L. H. Worodria W. O. Nahid P. Schorey J. S. (2014). Detection of *Mycobacterium tuberculosis* peptides in the exosomes of patients with active and latent *M. tuberculosis* infection using MRM-MS. PLoS One 9, e103811. 10.1371/journal.pone.0103811 25080351 PMC4117584

[B82] Kruh-Garcia N. A. Wolfe L. M. Dobos K. M. (2015). Deciphering the role of exosomes in tuberculosis. Tuberculosis (Edinb) 95, 26–30. 10.1016/j.tube.2014.10.010 25496995

[B83] Kulp A. J. Sun B. Ai T. Manning A. J. Orench-Rivera N. Schmid A. K. (2015). Genome-wide assessment of outer membrane vesicle production in *Escherichia coli* . PLoS One 10, e0139200. 10.1371/journal.pone.0139200 26406465 PMC4583269

[B84] Kumar A. Deep G. (2020). Exosomes in hypoxia-induced remodeling of the tumor microenvironment. Cancer Lett. 488, 1–8. 10.1016/j.canlet.2020.05.018 32473240

[B85] La Shu S. Yang Y. Allen C. L. Maguire O. Minderman H. Sen A. (2018). Metabolic reprogramming of stromal fibroblasts by melanoma exosome microRNA favours a pre-metastatic microenvironment. Sci. Rep. 8 (1), 12905. 10.1038/s41598-018-31323-7 30150674 PMC6110845

[B86] Laulagnier K. Motta C. Hamdi S. Roy S. Fauvelle F. Pageaux J. F. (2004). Mast cell- and dendritic cell-derived exosomes display a specific lipid composition and an unusual membrane organization. Biochem. J. 380, 161–171. 10.1042/BJ20031594 14965343 PMC1224152

[B87] Lázaro-Ibáñez E. Lunavat T. R. Jang S. C. Escobedo-Lucea C. Oliver-De La Cruz J. Siljander P. (2017). Distinct prostate cancer-related mRNA cargo in extracellular vesicle subsets from prostate cell lines. BMC Cancer 17, 92. 10.1186/s12885-017-3087-x 28143451 PMC5286827

[B88] Lee W. Nanou A. Rikkert L. Coumans F. A. W. Otto C. Terstappen L. W. M. M. (2018). Label-free prostate cancer detection by characterization of extracellular vesicles using Raman spectroscopy. Anal. Chem. 90, 11290–11296. 10.1021/acs.analchem.8b01831 30157378 PMC6170952

[B89] Levental I. Byfield F. J. Chowdhury P. Gai F. Baumgart T. Janmey P. A. (2009). Cholesterol-dependent phase separation in cell-derived giant plasma-membrane vesicles. Biochem. J. 424, 163–167. 10.1042/BJ20091283 19811449 PMC3118457

[B90] Levental K. R. Levental I. (2015). Giant plasma membrane vesicles: models for understanding membrane organization. Curr. Top. Membr. 75, 25–57. 10.1016/bs.ctm.2015.03.009 26015280

[B91] Levine A. J. Puzio-Kuter A. M. (2010). The control of the metabolic switch in cancers by oncogenes and tumor suppressor genes. Science 330, 1340–1344. 10.1126/science.1193494 21127244

[B92] Lin Q. Qu M. Zhou B. Patra H. K. Sun Z. Luo Q. (2019). Exosome-like nanoplatform modified with targeting ligand improves anti-cancer and anti-inflammation effects of imperialine. J. Control Release 311-312, 104–116. 10.1016/j.jconrel.2019.08.037 31484040

[B93] Llorente A. Skotland T. Sylvänne T. Kauhanen D. Róg T. Orłowski A. (2013). Molecular lipidomics of exosomes released by PC-3 prostate cancer cells. Biochim. Biophys. Acta 1831, 1302–1309. 10.1016/j.bbalip.2013.04.011 24046871

[B94] Lorent J. H. Diaz-Rohrer B. Lin X. Spring K. Gorfe A. A. Levental K. R. (2017). Structural determinants and functional consequences of protein affinity for membrane rafts. Nat. Commun. 8, 1219. 10.1038/s41467-017-01328-3 29089556 PMC5663905

[B95] Lucchetti D. Colella F. Perelli L. Ricciardi‐tenore C. Calapà F. Fiori M. E. (2020). CD147 promotes cell small extracellular vesicles release during colon cancer stem cells differentiation and triggers cellular changes in recipient cells. Cancers (Basel) 12. 10.3390/cancers12020260 PMC7072373 31973205

[B96] Lydic T. A. Townsend S. Adda C. G. Collins C. Mathivanan S. Reid G. E. (2015). Rapid and comprehensive 'shotgun' lipidome profiling of colorectal cancer cell derived exosomes. Methods 87, 83–95. 10.1016/j.ymeth.2015.04.014 25907253 PMC4615275

[B97] Ma L. Li Y. Peng J. Wu D. Zhao X. Cui Y. (2015). Discovery of the migrasome, an organelle mediating release of cytoplasmic contents during cell migration. Cell Res 25, 24–38. 10.1038/cr.2014.135 25342562 PMC4650581

[B98] Mantel P. Y. Hoang A. N. Goldowitz I. Potashnikova D. Hamza B. Vorobjev I. (2013). Malaria-infected erythrocyte-derived microvesicles mediate cellular communication within the parasite population and with the host immune system. Cell Host Microbe 13, 521–534. 10.1016/j.chom.2013.04.009 23684304 PMC3687518

[B99] Marcilla A. Martin-Jaular L. Trelis M. de Menezes-Neto A. Osuna A. Bernal D. (2014). Extracellular vesicles in parasitic diseases. J. Extracell. Vesicles 3, 25040. 10.3402/jev.v3.25040 25536932 PMC4275648

[B100] Marcilla A. Trelis M. Cortés A. Sotillo J. Cantalapiedra F. Minguez M. T. (2012). Extracellular vesicles from parasitic helminths contain specific excretory/secretory proteins and are internalized in intestinal host cells. PLoS One 7, e45974. 10.1371/journal.pone.0045974 23029346 PMC3454434

[B101] Marinko J. T. Kenworthy A. K. Sanders C. R. Kenworthy A. K. Sanders C. R. Sanders C. R. (2020). Peripheral myelin protein 22 preferentially partitions into ordered phase membrane domains. Proc. Natl. Acad. Sci. U. S. A. 117, 14168–14177. 10.1073/pnas.2000508117 32513719 PMC7322011

[B102] Marti M. Johnson P. J. (2016). Emerging roles for extracellular vesicles in parasitic infections. Curr. Opin. Microbiol. 32, 66–70. 10.1016/j.mib.2016.04.008 27208506 PMC6445373

[B103] Martin-Jaular L. Nakayasu E. S. Ferrer M. Almeida I. C. del Portillo H. A. (2011). Exosomes from Plasmodium yoelii-infected reticulocytes protect mice from lethal infections. PLoS One 6, e26588. 10.1371/journal.pone.0026588 22046311 PMC3202549

[B104] Mashburn L. M. Whiteley M. (2005). Membrane vesicles traffic signals and facilitate group activities in a prokaryote. Nature 437, 422–425. 10.1038/nature03925 16163359

[B105] Mashburn-Warren L. Howe J. Garidel P. Richter W. Steiniger F. Roessle M. (2008). Interaction of quorum signals with outer membrane lipids: insights into prokaryotic membrane vesicle formation. Mol. Microbiol. 69, 491–502. 10.1111/j.1365-2958.2008.06302.x 18630345 PMC2615190

[B106] Matsumura T. Sugimachi K. Iinuma H. Takahashi Y. Kurashige J. Sawada G. (2015). Exosomal microRNA in serum is a novel biomarker of recurrence in human colorectal cancer. Br. J. Cancer 113, 275–281. 10.1038/bjc.2015.201 26057451 PMC4506387

[B107] Mayers J. R. Audhya A. (2012). Vesicle formation within endosomes: an ESCRT marks the spot. Commun. Integr. Biol. 5, 50–56. 10.4161/cib.18208 22482010 PMC3291314

[B108] Melo S. A. Luecke L. B. Kahlert C. Fernandez A. F. Gammon S. T. Kaye J. (2015). Glypican-1 identifies cancer exosomes and detects early pancreatic cancer. Nature 523, 177–182. 10.1038/nature14581 26106858 PMC4825698

[B109] Minciacchi V. R. Freeman M. R. Di Vizio D. (2015). Extracellular vesicles in cancer: exosomes, microvesicles and the emerging role of large oncosomes. Semin. Cel Dev. Biol. 40, 41–51. 10.1016/j.semcdb.2015.02.010 PMC4747631 25721812

[B110] Minciacchi V. R. Spinelli C. Reis-Sobreiro M. Cavallini L. You S. Zandian M. (2017). MYC mediates large oncosome-induced fibroblast reprogramming in prostate cancer. Cancer Res. 77, 2306–2317. 10.1158/0008-5472.CAN-16-2942 28202510

[B111] Mogollón P. Díaz-Tejedor A. Algarín E. M. Paíno T. Garayoa M. Ocio E. M. (2019). Biological background of resistance to current standards of care in multiple myeloma. Cells 8 (11), 1432. 10.3390/cells8111432 PMC6912619 31766279

[B112] Morad G. Carman C. V. Hagedorn E. J. Perlin J. R. Zon L. I. Mustafaoglu N. (2019). Tumor-derived extracellular vesicles breach the intact blood-brain barrier via transcytosis. ACS Nano 13, 13853–13865. 10.1021/acsnano.9b04397 31479239 PMC7169949

[B113] Moyano A. L. Li G. Boullerne A. I. Feinstein D. L. Hartman E. Skias D. (2016). Sulfatides in extracellular vesicles isolated from plasma of multiple sclerosis patients. J. Neurosci. Res. 94, 1579–1587. 10.1002/jnr.23899 27557608

[B114] Mrowczynski O. D. Madhankumar A. B. Sundstrom J. M. Zhao Y. Kawasawa Y. I. Slagle-Webb B. (2018). Exosomes impact survival to radiation exposure in cell line models of nervous system cancer. Oncotarget 9 (90), 36083–36101. 10.18632/oncotarget.26300 30546829 PMC6281426

[B115] Nagakubo T. Nomura N. Toyofuku M. (2019). Cracking open bacterial membrane vesicles. Front. Microbiol. 10, 3026. 10.3389/fmicb.2019.03026 32038523 PMC6988826

[B116] Nguyen H. Q. Lee D. Kim Y. Paek M. Kim M. Jang K. S. (2019). Platelet factor 4 as a novel exosome marker in MALDI-MS analysis of exosomes from human serum. Anal. Chem. 91, 13297–13305. 10.1021/acs.analchem.9b04198 31549806

[B117] Ni J. Bucci J. Malouf D. Knox M. Graham P. Li Y. (2019). Exosomes in cancer radioresistance. Front. Oncol. 9, 869. 10.3389/fonc.2019.00869 31555599 PMC6742697

[B118] Nogueira P. M. de Menezes-Neto A. Borges V. M. Descoteaux A. Torrecilhas A. C. Xander P. (2020). Immunomodulatory properties of Leishmania extracellular vesicles during host-parasite interaction: differential activation of TLRs and NF-κB translocation by dermotropic and viscerotropic species. Front. Cel. Infect. Microbiol. 10, 380. 10.3389/fcimb.2020.00380 PMC7403210 32850481

[B119] O'Brien K. Breyne K. Ughetto S. Laurent L. C. Breakefield X. O. (2020). RNA delivery by extracellular vesicles in mammalian cells and its applications. Nat. Rev. Mol. Cel Biol. 21, 585–606. 10.1038/s41580-020-0251-y PMC7249041 32457507

[B120] Ohno S. Takanashi M. Sudo K. Ueda S. Ishikawa A. Matsuyama N. (2013). Systemically injected exosomes targeted to EGFR deliver antitumor microrna to breast cancer cells. Mol. Ther. 21, 185–191. 10.1038/mt.2012.180 23032975 PMC3538304

[B121] Osteikoetxea X. Balogh A. Szabó-Taylor K. Németh A. Szabó T. G. Pálóczi K. (2015). Improved characterization of EV preparations based on protein to lipid ratio and lipid properties. PLoS One 10, e0121184. 10.1371/journal.pone.0121184 25798862 PMC4370721

[B122] Parayath N. N. Padmakumar S. Amiji M. M. (2020). Extracellular vesicle-mediated nucleic acid transfer and reprogramming in the tumor microenvironment. Cancer Lett. 482, 33–43. 10.1016/j.canlet.2020.04.009 32289440

[B123] Parolini I. Federici C. Raggi C. Lugini L. Palleschi S. De Milito A. (2009). Microenvironmental pH is a key factor for exosome traffic in tumor cells. J. Biol. Chem. 284, 34211–34222. 10.1074/jbc.M109.041152 19801663 PMC2797191

[B124] Patel J. M. Vartabedian V. F. Bozeman E. N. Caoyonan B. E. Srivatsan S. Pack C. D. (2016). Plasma membrane vesicles decorated with glycolipid-anchored antigens and adjuvants via protein transfer as an antigen delivery platform for inhibition of tumor growth. Biomaterials 74, 231–244. 10.1016/j.biomaterials.2015.09.031 26461116 PMC4661141

[B125] Pérez-Cruz C. Carrión O. Delgado L. Martinez G. López-Iglesias C. Mercade E. (2013). New type of outer membrane vesicle produced by the gram-negative bacterium Shewanella vesiculosa M7T: implications for DNA content. Appl. Environ. Microbiol. 79, 1874–1881. 10.1128/AEM.03657-12 23315742 PMC3592255

[B126] Pérez-Cruz C. Delgado L. López-Iglesias C. Mercade E. (2015). Outer-inner membrane vesicles naturally secreted by gram-negative pathogenic bacteria. PLoS One 10. 10.1371/journal.pone.0116896 PMC4291224 25581302

[B127] Petanidis S. Domvri K. Porpodis K. Anestakis D. Freitag L. Hohenforst-Schmidt W. (2020). Inhibition of kras-derived exosomes downregulates immunosuppressive BACH2/GATA-3 expression via RIP-3 dependent necroptosis and miR-146/miR-210 modulation. Biomed. Pharmacother. 122, 109461. 10.1016/j.biopha.2019.109461 31918262

[B128] Petousis-Harris H. (2018). Impact of meningococcal group B OMV vaccines, beyond their brief. Hum. Vaccin. Immunother. 14, 1058–1063. 10.1080/21645515.2017.1381810 29048985 PMC5989908

[B129] Pi F. Binzel D. W. Lee T. J. Li Z. Sun M. Rychahou P. (2018). Nanoparticle orientation to control RNA loading and ligand display on extracellular vesicles for cancer regression. Nat. Nanotechnol. 13, 82–89. 10.1038/s41565-017-0012-z 29230043 PMC5762263

[B130] Pienimaeki-Roemer A. Kuhlmann K. Böttcher A. Konovalova T. Black A. Orsó E. (2015). Lipidomic and proteomic characterization of platelet extracellular vesicle subfractions from senescent platelets. Transfusion 55, 507–521. 10.1111/trf.12874 25332113

[B131] Podkalicka J. Blouin C. M. (2020). Gpmvs as a tool to study caveolin-interacting partners. Methods Mol. Biol. 2169, 81–88. 10.1007/978-1-0716-0732-9_8 32548821

[B132] Pope S. M. Lässer C. (2013). Toxoplasma gondii infection of fibroblasts causes the production of exosome-like vesicles containing a unique array of mRNA and miRNA transcripts compared to serum starvation. J. Extracell. Vesicles 2. 10.3402/jev.v2i0.22484 PMC3862870 24363837

[B133] Prados-Rosales R. Baena A. Martinez L. R. Luque-Garcia J. Kalscheuer R. Veeraraghavan U. (2011). Mycobacteria release active membrane vesicles that modulate immune responses in a TLR2-dependent manner in mice. J. Clin. Invest. 121, 1471–1483. 10.1172/JCI44261 21364279 PMC3069770

[B134] Prados-Rosales R. Carreño L. J. Batista-Gonzalez A. Baena A. Venkataswamy M. M. Xu J. (2014a). Mycobacterial membrane vesicles administered systemically in mice induce a protective immune response to surface compartments of mycobacterium tuberculosis. MBio 5, e01921. 10.1128/mBio.01921-14 25271291 PMC4196239

[B135] Prados-Rosales R. Weinrick B. C. Piqué D. G. Jacobs W. R. Casadevall A. Rodriguez G. M. (2014b). Role for mycobacterium tuberculosis membrane vesicles in iron acquisition. J. Bacteriol. 196, 1250–1256. 10.1128/JB.01090-13 24415729 PMC3957709

[B136] Raghunathan K. Kenworthy A. K. (20182018–2031). Dynamic pattern generation in cell membranes: current insights into membrane organization. Biochim. Biophys. Acta Biomembr 1860, 2018. 10.1016/j.bbamem.2018.05.002 PMC6234104 29752898

[B137] Ramakrishnan V. Xu B. Akers J. Nguyen T. Ma J. Dhawan S. (2020). Radiation-induced extracellular vesicle (EV) release of miR-603 promotes IGF1-mediated stem cell state in glioblastomas. EBioMedicine 55, 102736. 10.1016/j.ebiom.2020.102736 32361246 PMC7195524

[B138] Regev-Rudzki N. Wilson D. W. Carvalho T. G. Sisquella X. Coleman B. M. Rug M. (2013). Cell-cell communication between malaria-infected red blood cells via exosome-like vesicles. Cell 153, 1120–1133. 10.1016/j.cell.2013.04.029 23683579

[B139] Reid M. Kong M. (2013). Dealing with hunger: metabolic stress responses in tumors. J. Carcinog. 12, 17. 10.4103/1477-3163.119111 24227992 PMC3816312

[B140] Resch U. Tsatsaronis J. A. Le Rhun A. Stübiger G. Rohde M. Kasvandik S. (2016). A two-component regulatory system impacts extracellular membrane-derived vesicle production in group a streptococcus. MBio 7. 10.1128/mBio.00207-16 PMC5090034 27803183

[B141] Ribeiro K. S. Vasconcellos C. I. Soares R. P. Mendes M. T. Ellis C. C. Aguilera-Flores M. (2018). Proteomic analysis reveals different composition of extracellular vesicles released by two Trypanosoma cruzi strains associated with their distinct interaction with host cells. J. Extracell. Vesicles 7, 1463779. 10.1080/20013078.2018.1463779 29696081 PMC5912195

[B142] Ridder K. Keller S. Dams M. Rupp A. K. Schlaudraff J. Del Turco D. (2014). Extracellular vesicle-mediated transfer of genetic information between the hematopoietic system and the brain in response to inflammation. Plos Biol. 12, e1001874. 10.1371/journal.pbio.1001874 24893313 PMC4043485

[B143] Royo F. Gil-Carton D. Gonzalez E. Mleczko J. Palomo L. Perez-Cormenzana M. (2019). Differences in the metabolite composition and mechanical properties of extracellular vesicles secreted by hepatic cellular models. J. Extracell. Vesicles 8. 10.1080/20013078.2019.1575678 PMC6374943 30788084

[B144] Sancho-Albero M. Rubio-Ruiz B. Pérez-López A. M. Sebastián V. Martín-Duque P. Arruebo M. (2019). Cancer-derived exosomes loaded with ultrathin palladium nanosheets for targeted bioorthogonal catalysis. Nat. Catal. 2, 864–872. 10.1038/s41929-019-0333-4 31620674 PMC6795537

[B145] Schaar V. De Vries S. P. Perez Vidakovics M. L. Bootsma H. J. Larsson L. Hermans P. W. (2011a). Multicomponent Moraxella catarrhalis outer membrane vesicles induce an inflammatory response and are internalized by human epithelial cells. Cell. Microbiol. 13, 432–449. 10.1111/j.1462-5822.2010.01546.x 21044239

[B146] Schaar V. Nordström T. Mörgelin M. Riesbeck K. (2011b). Moraxella catarrhalis outer membrane vesicles carry β-lactamase and promote survival of Streptococcus pneumoniae and Haemophilus influenzae by inactivating amoxicillin. Antimicrob. Agents Chemother. 55, 3845–3853. 10.1128/AAC.01772-10 21576428 PMC3147650

[B147] Schaefer A. L. Taylor T. A. Beatty J. T. Greenberg E. P. (2002). Long-chain acyl-homoserine lactone quorum-sensing regulation of Rhodobacter capsulatus gene transfer agent production. J. Bacteriol. 184, 6515–6521. 10.1128/JB.184.23.6515-6521.2002 12426339 PMC135431

[B148] Schatz D. Rosenwasser S. Malitsky S. Wolf S. G. Feldmesser E. Vardi A. (2017). Communication via extracellular vesicles enhances viral infection of a cosmopolitan alga. Nat. Microbiol. 2, 1485–1492. 10.1038/s41564-017-0024-3 28924189

[B149] Schild S. Nelson E. J. Camilli A. (2008). Immunization with *Vibrio cholerae* outer membrane vesicles induces protective immunity in mice. Infect. Immun. 76, 4554–4563. 10.1128/IAI.00532-08 18678672 PMC2546833

[B150] Schneider F. Waithe D. Clausen M. P. Galiani S. Koller T. Ozhan G. (2017). Diffusion of lipids and GPI-anchored proteins in actin-free plasma membrane vesicles measured by STED-FCS. Mol. Biol. Cel 28, 1507–1518. 10.1091/mbc.E16-07-0536 PMC5449149 28404749

[B151] Schorey J. S. Bhatnagar S. (2008). Exosome function: from tumor immunology to pathogen biology. Traffic 9, 871–881. 10.1111/j.1600-0854.2008.00734.x 18331451 PMC3636814

[B152] Schroeder R. London E. Brown D. (1994). Interactions between saturated acyl chains confer detergent resistance on lipids and glycosylphosphatidylinositol (GPI)-anchored proteins: GPI-anchored proteins in liposomes and cells show similar behavior. Proc. Natl. Acad. Sci. USA 91, 12130–12134. 10.1073/pnas.91.25.12130 7991596 PMC45390

[B153] Scott R. E. Maercklein P. B. (1979). Plasma membrane vesiculation in 3T3 and SV3T3 cells. II. Factors affecting the process of vesiculation. J. Cel Sci. 35, 245–252. 10.1242/jcs.35.1.245 422673

[B154] Sengupta P. Hammond A. Holowka D. Baird B. (2008). Structural determinants for partitioning of lipids and proteins between coexisting fluid phases in giant plasma membrane vesicles. Biochim. Biophys. Acta 1778, 20–32. 10.1016/j.bbamem.2007.08.028 17936718 PMC2679899

[B155] Sengupta P. Seo A. Y. Pasolli H. A. Song Y. E. Johnson M. C. Lippincott-Schwartz J. (2019). A lipid-based partitioning mechanism for selective incorporation of proteins into membranes of HIV particles. Nat. Cel Biol. 21, 452–461. 10.1038/s41556-019-0300-y 30936472

[B156] Serruto D. Bottomley M. J. Ram S. Giuliani M. M. Rappuoli R. (2012). The new multicomponent vaccine against meningococcal serogroup B, 4CMenB: immunological, functional and structural characterization of the antigens. Vaccine 30 Suppl 2, B87. 10.1016/j.vaccine.2012.01.033 22607904 PMC3360877

[B157] Sezgin E. Waithe D. Bernardino De La Serna J. Eggeling C. (2015). Spectral imaging to measure heterogeneity in membrane lipid packing. ChemPhysChem 16, 1387–1394. 10.1002/cphc.201402794 25755090 PMC4539592

[B158] Silverman J. M. Chan S. K. Robinson D. P. Dwyer D. M. Nandan D. Foster L. J. (2008). Proteomic analysis of the secretome of Leishmania donovani. Genome Biol. 9, R35. 10.1186/gb-2008-9-2-r35 18282296 PMC2374696

[B159] Silverman J. M. Clos J. de'Oliveira C. C. Shirvani O. Fang Y. Wang C. (2010). An exosome-based secretion pathway is responsible for protein export from Leishmania and communication with macrophages. J. Cel Sci. 123, 842–852. 10.1242/jcs.056465 20159964

[B160] Singhto N. Vinaiphat A. Thongboonkerd V. (2019). Discrimination of urinary exosomes from microvesicles by lipidomics using thin layer liquid chromatography (TLC) coupled with MALDI-TOF mass spectrometry. Sci. Rep. 9, 13834. 10.1038/s41598-019-50195-z 31554842 PMC6761130

[B161] Skinkle A. D. Levental K. R. Levental I. (2020). Cell-derived plasma membrane vesicles are permeable to hydrophilic macromolecules. Biophys. J. 118, 1292–1300. 10.1016/j.bpj.2019.12.040 32053777 PMC7091462

[B162] Skotland T. Ekroos K. Kauhanen D. Simolin H. Seierstad T. Berge V. (2017a). Molecular lipid species in urinary exosomes as potential prostate cancer biomarkers. Eur. J. Cancer 70, 122–132. 10.1016/j.ejca.2016.10.011 27914242

[B163] Skotland T. Hessvik N. P. Sandvig K. Llorente A. (2019). Exosomal lipid composition and the role of ether lipids and phosphoinositides in exosome biology. J. Lipid Res. 60, 9–18. 10.1194/jlr.R084343 30076207 PMC6314266

[B164] Skotland T. Sandvig K. Llorente A. (2017b). Lipids in exosomes: current knowledge and the way forward. Prog. Lipid Res. 66, 30–41. 10.1016/j.plipres.2017.03.001 28342835

[B165] Slaughter M. J. Shanle E. K. McFadden A. W. Hollis E. S. Suttle L. E. Strahl B. D. (2018). PBRM1 bromodomains variably influence nucleosome interactions and cellular function. J. Biol. Chem. 293, 13592–13603. 10.1074/jbc.RA118.003381 29986887 PMC6120218

[B166] Song Z. Li B. Zhang Y. Li R. Ruan H. Wu J. (2020). Outer membrane vesicles of *Helicobacter pylori* 7.13 as adjuvants promote protective efficacy against *Helicobacter pylori* infection. Front. Microbiol. 11. 10.3389/fmicb.2020.01340 PMC7358646 32733396

[B167] Steinkühler J. Sezgin E. Urbančič I. Eggeling C. Dimova R. (2019). Mechanical properties of plasma membrane vesicles correlate with lipid order, viscosity and cell density. Commun. Biol. 2, 1–8. 10.1038/s42003-019-0583-3 31531398 PMC6744421

[B168] Stevenson T. C. Cywes-Bentley C. Moeller T. D. Weyant K. B. Putnam D. Chang Y. F. (2018). Immunization with outer membrane vesicles displaying conserved surface polysaccharide antigen elicits broadly antimicrobial antibodies. Proc. Natl. Acad. Sci. USA 115, E3106–E3115. 10.1073/pnas.1718341115 29555731 PMC5889644

[B169] Sun D. Zhuang X. Xiang X. Liu Y. Zhang S. Liu C. (2010). A novel nanoparticle drug delivery system: the anti-inflammatory activity of curcumin is enhanced when encapsulated in exosomes. Mol. Ther. 18, 1606–1614. 10.1038/mt.2010.105 20571541 PMC2956928

[B170] Sun Y. Saito K. Saito Y. (2019). Lipid profile characterization and lipoprotein comparison of extracellular vesicles from human plasma and serum. Metabolites 9. 10.3390/metabo9110259 PMC6918450 31683897

[B171] Sung J. S. Kang C. W. Kang S. Jang Y. Chae Y. C. Kim B. G. (2020). ITGB4-mediated metabolic reprogramming of cancer-associated fibroblasts. Oncogene 39 (3), 664–676. 10.1038/s41388-019-1014-0 31534187

[B172] Szatmári T. Hargitai R. Sáfrány G. Lumniczky K. (2019). Extracellular vesicles in modifying the effects of ionizing radiation. Int. J. Mol. Sci. 20 (22), 5527. 10.3390/ijms20225527 PMC6888126 31698689

[B173] Tang T. T. Lv L. L. Wang B. Cao J. Y. Feng Y. Li Z. L. (2019). Employing macrophage-derived microvesicle for kidney-targeted delivery of dexamethasone: an efficient therapeutic strategy against renal inflammation and fibrosis. Theranostics 9, 4740–4755. 10.7150/thno.33520 31367254 PMC6643445

[B174] Tanziela T. Shaikh S. Jiang H. Lu Z. Wang X. (2020). Efficient encapsulation of biocompatible nanoparticles in exosomes for cancer theranostics. Nano Today 35. 10.1016/j.nantod.2020.100964

[B175] Tao L. Zhou J. Yuan C. Zhang L. Li D. Si D. (2019). Metabolomics identifies serum and exosomes metabolite markers of pancreatic cancer. Metabolomics 15, 86. 10.1007/s11306-019-1550-1 31147790

[B176] Thoma J. Burmann B. M. (2020). High-resolution in situ NMR spectroscopy of bacterial envelope proteins in outer membrane vesicles. Biochemistry 59, 1656–1660. 10.1021/acs.biochem.9b01123 32233422 PMC7310948

[B177] Thoma J. Manioglu S. Kalbermatter D. Bosshart P. D. Fotiadis D. Müller D. J. (2018). Protein-enriched outer membrane vesicles as a native platform for outer membrane protein studies. Commun. Biol. 1, 23. 10.1038/s42003-018-0027-5 30271910 PMC6123736

[B178] Tian Y. Li S. Song J. Ji T. Zhu M. Anderson G. J. (2014). A doxorubicin delivery platform using engineered natural membrane vesicle exosomes for targeted tumor therapy. Biomaterials 35, 2383–2390. 10.1016/j.biomaterials.2013.11.083 24345736

[B179] Todorova D. Simoncini S. Lacroix R. Sabatier F. Dignat-George F. (2017). Extracellular vesicles in angiogenesis. Circ. Res. 120, 1658. 10.1161/CIRCRESAHA.117.309681 28495996 PMC5426696

[B180] Toledo A. Huang Z. Coleman J. L. London E. Benach J. L. (2018). Lipid rafts can form in the inner and outer membranes of Borrelia burgdorferi and have different properties and associated proteins. Mol. Microbiol. 108, 63–76. 10.1111/mmi.13914 29377398 PMC5867248

[B181] Toyofuku M. Morinaga K. Hashimoto Y. Uhl J. Shimamura H. Inaba H. (2017). Membrane vesicle-mediated bacterial communication. ISME J. 11, 1504–1509. 10.1038/ismej.2017.13 28282039 PMC5437348

[B182] Toyofuku M. Nomura N. Eberl L. (2019). Types and origins of bacterial membrane vesicles. Nat. Rev. Microbiol. 17, 13–24. 10.1038/s41579-018-0112-2 30397270

[B183] Trajkovic K. Hsu C. Chiantia S. Rajendran L. Wenzel D. Wieland F. (2008). Ceramide triggers budding of exosome vesicles into multivesicular endosomes. Science 319, 1244–1247. 10.1126/science.1153124 18309083

[B184] Tran P. H. L. Xiang D. Tran T. T. D. Yin W. Zhang Y. Kong L. (2020). Exosomes and nanoengineering: a match made for precision therapeutics. Adv. Mater. 32. 10.1002/adma.201904040 31531916

[B185] Twu O. de Miguel N. Lustig G. Stevens G. C. Vashisht A. A. Wohlschlegel J. A. (2013). Trichomonas vaginalis exosomes deliver cargo to host cells and mediate host∶parasite interactions. Plos Pathog. 9, e1003482. 10.1371/journal.ppat.1003482 23853596 PMC3708881

[B186] Valkonen S. Holopainen M. Colas R. A. Impola U. Dalli J. Käkelä R. (2019). Lipid mediators in platelet concentrate and extracellular vesicles: molecular mechanisms from membrane glycerophospholipids to bioactive molecules. Biochim. Biophys. Acta Mol. Cel Biol Lipids 1864, 1168–1182. 10.1016/j.bbalip.2019.03.011 30980920

[B187] Van Meer G. Voelker D. R. Feigenson G. W. (2008). Membrane lipids: where they are and how they behave. Nat. Rev. Mol. Cel Biol. 9, 112–124. 10.1038/nrm2330 PMC2642958 18216768

[B188] Vigano S. Alatzoglou D. Irving M. Ménétrier-Caux C. Caux C. Romero P. (2019). Targeting adenosine in cancer immunotherapy to enhance T-Cell function. Front. Immunol. 10, 925. 10.3389/fimmu.2019.00925 31244820 PMC6562565

[B189] Wagner T. Joshi B. Janice J. Askarian F. Škalko-Basnet N. Hagestad O. C. (2018). Enterococcus faecium produces membrane vesicles containing virulence factors and antimicrobial resistance related proteins. J. Proteomics 187, 28–38. 10.1016/j.jprot.2018.05.017 29857065

[B190] Wan L. Xia T. Du Y. Liu J. Xie Y. Zhang Y. (2019). Exosomes from activated hepatic stellate cells contain GLUT1 and PKM2: a role for exosomes in metabolic switch of liver nonparenchymal cells. FASEB J. 33 (7), 8530–8542. 10.1096/fj.201802675R 30970216

[B191] Wang B. Wang X. Hou D. Huang Q. Zhan W. Chen C. (2019). Exosomes derived from acute myeloid leukemia cells promote chemoresistance by enhancing glycolysis-mediated vascular remodeling. J. Cel. Physiol. 234 (7), 10602–10614. 10.1002/jcp.27735 30417360

[B192] Wang D. Yao Y. He J. Zhong X. Li B. Rao S. (2020). Engineered cell-derived microparticles Bi2Se3/DOX@MPs for imaging guided synergistic photothermal/low-dose chemotherapy of cancer. Adv. Sci. 7, 1901293. 10.1002/advs.201901293 PMC7001653 32042550

[B193] Wang F. Li L. Piontek K. Sakaguchi M. Selaru F. M. (2018). Exosome miR-335 as a novel therapeutic strategy in hepatocellular carcinoma. Hepatology 67, 940–954. 10.1002/hep.29586 29023935 PMC5826829

[B194] Wang H. Jiang D. Li W. Xiang X. Zhao J. Yu B. (2019). Evaluation of serum extracellular vesicles as noninvasive diagnostic markers of glioma. Theranostics 9, 5347–5358. 10.7150/thno.33114 31410219 PMC6691576

[B195] Wang J. Wang Y. Tang L. Garcia R. C. (2019). Extracellular vesicles in mycobacterial infections: their potential as molecule transfer vectors. Front. Immunol. 10. 10.3389/fimmu.2019.01929 PMC6703136 31474995

[B196] Wang X. Cheng K. Zhang G. Jia Z. Yu Y. Guo J. (2020). Enrichment of CD44 in exosomes from breast cancer cells treated with doxorubicin promotes chemoresistance. Front. Oncol. 10, 960. 10.3389/fonc.2020.00960 32760666 PMC7373100

[B197] Wang Y. H. Bucki R. Janmey P. A. (2016). Cholesterol-dependent phase-demixing in lipid bilayers as a switch for the activity of the phosphoinositide-binding cytoskeletal protein gelsolin. Biochemistry 55, 3361–3369. 10.1021/acs.biochem.5b01363 27224309 PMC4975948

[B198] Waters C. M. Bassler B. L. (2005). Quorum sensing: cell-to-cell communication in bacteria. Annu. Rev. Cel Dev. Biol. 21, 319–346. 10.1146/annurev.cellbio.21.012704.131001 16212498

[B199] Wiklander O. P. Nordin J. Z. O'Loughlin A. Gustafsson Y. Corso G. Mäger I. (2015). Extracellular vesicle *in vivo* biodistribution is determined by cell source, route of administration and targeting. J. Extracell. Vesicles 4, 26316–26413. 10.3402/jev.v4.26316 25899407 PMC4405624

[B200] Wiklander O. P. B. Brennan M. Lötvall J. Breakefield X. O. Andaloussi S. E. L. (2019). Advances in therapeutic applications of extracellular vesicles. Sci. Transl. Med. 11 (492), 1–13. 10.3402/jev.v4.26316 PMC7104415 31092696

[B201] Worch R. Petrášek Z. Schwille P. Weidemann T. (2017). Diffusion of single-pass transmembrane receptors: from the plasma membrane into giant liposomes. J. Membr. Biol. 250, 393–406. 10.1007/s00232-016-9936-8 27826635 PMC5579168

[B202] Wu Q. Sun S. Li Z. Yang Q. Li B. Zhu S. (2018). Tumour-originated exosomal miR-155 triggers cancer-associated cachexia to promote tumour progression. Mol. Cancer 17 (1), 155. 10.1186/s12943-018-0899-5 30359265 PMC6201501

[B203] Wubbolts R. Leckie R. S. Veenhuizen P. T. Schwarzmann G. Möbius W. Hoernschemeyer J. (2003). Proteomic and biochemical analyses of human B cell-derived exosomes. Potential implications for their function and multivesicular body formation. J. Biol. Chem. 278, 10963–10972. 10.1074/jbc.M207550200 12519789

[B204] Xing Y. Cheng Z. Wang R. Lv C. James T. D. Yu F. (2020). Analysis of extracellular vesicles as emerging theranostic nanoplatforms. Coord. Chem. Rev. 424. 10.1016/j.ccr.2020.213506

[B205] Xiong F. Ling X. Chen X. Chen J. Tan J. Cao W. (2019). Pursuing specific chemotherapy of orthotopic breast cancer with lung metastasis from docking nanoparticles driven by bioinspired exosomes. Nano Lett. 19, 3256–3266. 10.1021/acs.nanolett.9b00824 30965009

[B206] Xu J. Wang Y. Hsu C. Y. Gao Y. Meyers C. A. Chang L. (2019). Human perivascular stem cell-derived extracellular vesicles mediate bone repair. Elife 8. 10.7554/eLife.48191 PMC6764819 31482845

[B207] Yang B. Chen Y. Shi J. (2019). Exosome biochemistry and advanced nanotechnology for next-generation theranostic platforms. Adv. Mater. 31. 10.1002/adma.201802896 30126052

[B208] Yang J. S. Lee J. C. Byeon S. K. Rha K. H. Moon M. H. (2017). Size dependent lipidomic analysis of urinary exosomes from patients with prostate cancer by flow field-flow fractionation and nanoflow liquid chromatography-tandem mass spectrometry. Anal. Chem. 89, 2488–2496. 10.1021/acs.analchem.6b04634 28192938

[B209] Yang S. Che S. P. Kurywchak P. Tavormina J. L. Gansmo L. B. Correa de Sampaio P. (2017). Detection of mutant KRAS and TP53 DNA in circulating exosomes from healthy individuals and patients with pancreatic cancer. Cancer Biol. Ther. 18, 158–165. 10.1080/15384047.2017.1281499 28121262 PMC5389423

[B210] Yang S. T. Kiessling V. Tamm L. K. (2016). Line tension at lipid phase boundaries as driving force for HIV fusion peptide-mediated fusion. Nat. Commun. 7, 11401. 10.1038/ncomms11401 27113279 PMC4853434

[B211] Yang S. T. Kreutzberger A. J. B. Kiessling V. Ganser-Pornillos B. K. White J. M. Tamm L. K. (2017). HIV virions sense plasma membrane heterogeneity for cell entry. Sci. Adv. 3, e1700338. 10.1126/sciadv.1700338 28782011 PMC5489272

[B212] Yekula A. Yekula A. Muralidharan K. Kang K. Carter B. S. Balaj L. (2019b). Extracellular vesicles in glioblastoma tumor microenvironment. Front. Immunol. 10, 3137. 10.3389/fimmu.2019.03137 32038644 PMC6990128

[B213] Yekula A. Taylor A. Beecroft A. Kang K. M. Small J. L. Muralidharan K. (2020a). The role of extracellular vesicles in acquisition of resistance to therapy in glioblastomas. Cancer Drug Resist. 20 (20), 5107. 10.20517/cdr.2020.61 PMC9019190 35582008

[B214] Ying Y. C. Hao S. B. Tan T. M. C. Mattmann M. E. Geske G. D. Igarashi J. (2007). Control of quorum sensing by a Burkholderia pseudomallei multidrug efflux pump. J. Bacteriol. 189, 4320–4324. 10.1128/JB.00003-07 17384185 PMC1913402

[B215] Yong T. Zhang X. Bie N. Zhang H. Zhang X. Li F. (2019). Tumor exosome-based nanoparticles are efficient drug carriers for chemotherapy. Nat. Commun. 10, 3838. 10.1038/s41467-019-11718-4 31444335 PMC6707218

[B216] Zhang D. Qin X. Wu T. Qiao Q. Song Q. Zhang Z. (2019). Extracellular vesicles based self-grown gold nanopopcorn for combinatorial chemo-photothermal therapy. Biomaterials 197, 220–228. 10.1016/j.biomaterials.2019.01.024 30669014

[B217] Zhang H. Freitas D. Kim H. S. Fabijanic K. Li Z. Chen H. (2018). Identification of distinct nanoparticles and subsets of extracellular vesicles by asymmetric flow field-flow fractionation. Nat. Cel Biol. 20, 332–343. 10.1038/s41556-018-0040-4 PMC5931706 29459780

[B218] Zhang J. Lu S. Zhou Y. Meng K. Chen Z. Cui Y. (2017). Motile hepatocellular carcinoma cells preferentially secret sugar metabolism regulatory proteins via exosomes. Proteomics 17. 10.1002/pmic.201700103 28590090

[B219] Zhao J. Liu C. Li Y. Ma Y. Deng J. Li L. (2020). Thermophoretic detection of exosomal microRNAs by nanoflares. J. Am. Chem. Soc. 142, 4996–5001. 10.1021/jacs.9b13960 32134270

[B220] Zhao K. Deng X. He C. Yue B. Wu M. (2013). *Pseudomonas aeruginosa* outer membrane vesicles modulate host immune responses by targeting the toll-like receptor 4 signaling pathway. Infect. Immun. 81, 4509–4518. 10.1128/IAI.01008-13 24082079 PMC3837971

